# Graph Neural Network Driven Exploration of Non‐Precious Metal Catalysts for Air‐to‐Ammonia Conversion

**DOI:** 10.1002/adma.202509915

**Published:** 2025-08-01

**Authors:** Chengyi Zhang, Xiaoli Ge, Zihao Jiao, Mengyao Chang, Chuang Zhao, Qingsong Hua, Zhaoqiang Li, Geoffrey I.N. Waterhouse, Yuguang C. Li, Ziyun Wang

**Affiliations:** ^1^ School of Chemical Sciences University of Auckland SCIENCE CENTRE 302 ‐ Bldg 302, 23 SYMONDS ST, AUCKLAND CENTRAL, AUCKLAND, 1010, New Zealand Auckland 1010 New Zealand; ^2^ Department of Chemistry University at Buffalo State University of New York New York 14260 USA; ^3^ Department of Physics Faculty of Arts and Sciences Beijing Normal University Zhuhai 519085 China

**Keywords:** AI‐driven materials exploration, air‐to‐ammonia conversion, graph neural networks, high‐throughput screening, microkinetic modeling, non‐noble catalysts design

## Abstract

Efficient ammonia production directly from the air with minimal energy consumption remains one of the most challenging and ambitious scientific goals. NH_2_OH has proven to be a promising stable intermediate in producing NH_3_, with the direct generation of NH_3_ from air achieved by coupling a continuous flow plasma reactor with an electrolyzer. However, the requirement of noble metal‐doped Cu alloys as the cathode catalyst limits the scalability and cost‐effectiveness of the coupled plasma‐electrochemical system. In this work, graph neural networks, density functional theory calculations, and microkinetic modeling are combined to exhaustively explore the catalytic properties of all experimentally accessible alloy phases for NH_3_ production, ultimately identifying the non‐noble CuMnSb system as highly active for the conversion of air to NH_3_. The experiments confirm an ammonia production rate of 28.47 mg h^−1^ cm^−2^ in a coupled plasma‐electrolyser system. Such a finding confirms the future of machine learning and microkinetic theory in guiding the experimental exploration that transcends the constraints of conventional methods.

## Graphical abstract

A machine learning‐driven microkinetic framework, based on graph neural networks is developed, for screening non‐noble catalysts in NH_2_OH hydrogenation. This approach enables the discovery of efficient catalysts for plasma–electrochemical ammonia synthesis directly from air under ambient conditions. CuMnSb is identified as a top‐performing, sustainable candidate among all synthesizable alloy phases.

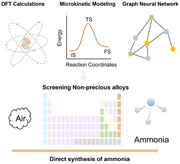

## 1 Introduction

Ammonia, as an essential chemical in fertilizer production, plays a vital role in feeding the global population. Currently, ammonia production predominantly relies on the Haber–Bosch process, which faces several serious drawbacks, such as high energy consumption, significant greenhouse gas emissions, fossil fuel dependence, high operational costs, safety concerns, and negative environmental impacts. The most critical issue is the requirement for hydrogen, which leads to a large amount of greenhouse gas emissions each year.^[^ ^1^ ^]^ One of the most promising alternative approaches is the electrochemical nitrogen reduction reaction (NRR), wherein the hydrogen source is water, significantly lowering energy consumption and greenhouse gas emissions.^[^ ^2^ ^]^ However, current NRR technologies face two significant challenges: low production rates and faradaic efficiencies (FE). These issues arise from the competing hydrogen evolution reaction (HER) and the high stability of the triple bond in the dinitrogen molecule.^[^ ^1^, ^3^ ^]^ To overcome these bottlenecks, researchers have put great effort into Li‐mediated nitrogen reduction^[^ ^4^ ^]^ and nitrate/nitrite electrochemical reduction.^[^ ^5^ ^]^

Recently, non‐thermal plasma has emerged as a promising strategy for breaking dinitrogen triple bonds. Coupling air plasma reactors that generate NO_x_H_y_ species with an electrolyzer can significantly boost the FE toward NH_3_.^[^ ^6^ ^]^ In this two‐step approach, N_2_ molecules are initially broken down into more reactive intermediates, serving as the reactants for the following electrochemical process. This strategy dramatically lowers overall energy consumption and enhances the NH_3_ production efficiency. Our recent work has highlighted the critical role of key intermediates, particularly NH_2_OH, during electrocatalysis, achieving the efficient direct conversion of air into ammonia by the plasma‐electrocatalysis process within a straightforward setup.^[^ ^7^ ^]^ It promises to eliminate the drawbacks of the Haber–Bosch process whilst enabling clean, efficient, low‐cost, intelligent, and fully automated on‐demand agricultural fertilizer production. However, the current reliance on noble metal‐doped Cu alloys for NO_x_H_y_ conversion to NH_3_ greatly hinders the scalability of plasma‐electrolyser systems. Identifying non‐noble catalysts is the crucial step for the scalability of such air‐to‐ammonia systems.

Previously, screening and discovering new catalytic materials primarily relied on experimental trial‐and‐error methods.^[^ ^8^ ^]^ However, such methods were time‐consuming and resource‐intensive, which greatly limited the pace of innovation. With the progress of computational technologies, density functional theory (DFT) has become a reliable substitute for materials screening. DFT offers a way to predict material properties with quantum mechanical accuracy, significantly reducing the reliance on experiments. However, despite its reliability, DFT calculations are computationally expensive, mainly when dealing with large‐scale material databases or complex systems, making high‐throughput screening challenging.^[^ ^9^ ^]^ Machine learning (ML) has emerged as a transformative tool in material science, fundamentally providing an approach to predicting material properties. ML can achieve exceptional speed and accuracy in predictions by leveraging existing data and reducing reliance on time‐intensive experimental or computational methods.^[^ ^10^ ^]^ Among ML approaches, graph neural networks (GNNs) have demonstrated exceptional efficiency for modeling materials, as they represent atomic structures as graphs, with atoms as nodes and bonds as edges. This representation allows GNNs to naturally capture complex relationships within material structures, providing fundamental insights into their properties.^[^ ^11^ ^]^

The current work aims to apply GNNs to screen catalysts for NH_2_OH generation, the key intermediates from air, for reduction to NH_3_. We first established the Brønsted–Evans–Polanyi (BEP) relationship^[^ ^12^ ^]^ between the Gibbs energy change (ΔG) and activation energy barrier on various metals for converting ^*^NH_2_OH to ^*^NH_2_, then ^*^NH_2_ to NH_3_. We then identified the scaling relationship between the adsorption energy of ^*^NH_2_ and ^*^NH_2_OH, allowing each ΔG to be expressed in adsorption energies toward NH_2_ and H. Furthermore, a projection from the GNN energy space toward the DFT energy space was established, facilitating large‐scale materials screening with improved accessibility and efficiency. Microkinetic modeling identified the optimal area for converting NH_2_OH toward NH_3_ in the ^*^NH_2_ adsorption energy vs ^*^H adsorption energy plot.^[^ ^13^ ^]^ Using GNNs, we obtained the adsorption energy toward ^*^NH_2_ and ^*^H of all experiment‐verified alloys (all non‐toxic and non‐radioactive experiment‐validated alloys (over 2000 alloys and over 40 000 configurations)) in the Materials Project database.^[^ ^14^ ^]^ This enabled the screening of a series of candidates for further evaluation. Detailed DFT calculations were subsequently conducted to filter the candidates regarding formation energy per atom and the activation energy barrier of the rate‐determining step (RDS), ultimately identifying CuMnSb as the most promising catalyst for converting NH_2_OH to NH_3_ in terms of activity and stability. Experimental validation in a coupled plasma‐electrolyzer system confirmed the simulation results, delivering an impressive ammonia production rate of 28.47 mg h^−1^ cm^−2^ for the air‐to‐ammonia process.

## 2 Results and Discussion

We start by presenting a detailed explanation of our theoretical framework to understand our work comprehensively. Using DFT, we evaluated the ΔG and activation energy barrier of each step of NH_2_OH reduction on the surface of Fe, Cu, Ni, Co, Os, Ag, Au, Rh, Ir, and Pd. These elements were selected to provide a broad pool of metallic candidates, ensuring the findings broadly apply to various material systems. At the same time, most of them are thermodynamically resistant to bulk oxide formation in water at 298 K and U = 0 V vs SHE.^[^ ^15^ ^]^

### 2.1 Materials Filtering Process

We first investigated the BEP relationship between the reaction barriers and ΔG for the hydrogenation steps involved in NH_2_OH reduction. Specifically, we analyzed four distinct reactions: converting ^*^NH_2_OH to ^*^NH_2_ and ^*^NH_2_ to NH_3_ via Langmuir–Hinshelwood (LH) and Eley–Rideal (ER) mechanisms. As the hydrogenation of NH_2_OH on various metal surfaces typically proceeds via a proton‐coupled electron transfer (PCET) mechanism, in which the proton and electron are transferred simultaneously. This type of reaction is a prototypical case where the computational hydrogen electrode (CHE) model can be reliably applied. In PCET steps, the free energy of the transition state and final state varies approximately linearly with the applied electrode potential, allowing the CHE approach to capture potential‐dependent energy profiles without the need for explicit charge control.^[^ ^16^ ^]^ Therefore, in this work, we employ the CHE model to describe the potential‐dependent energetics of the hydrogenation of NH_2_OH across different metal surfaces, including the changes in free energy of intermediates and transition states as a function of applied potential.

**Figure 1:**
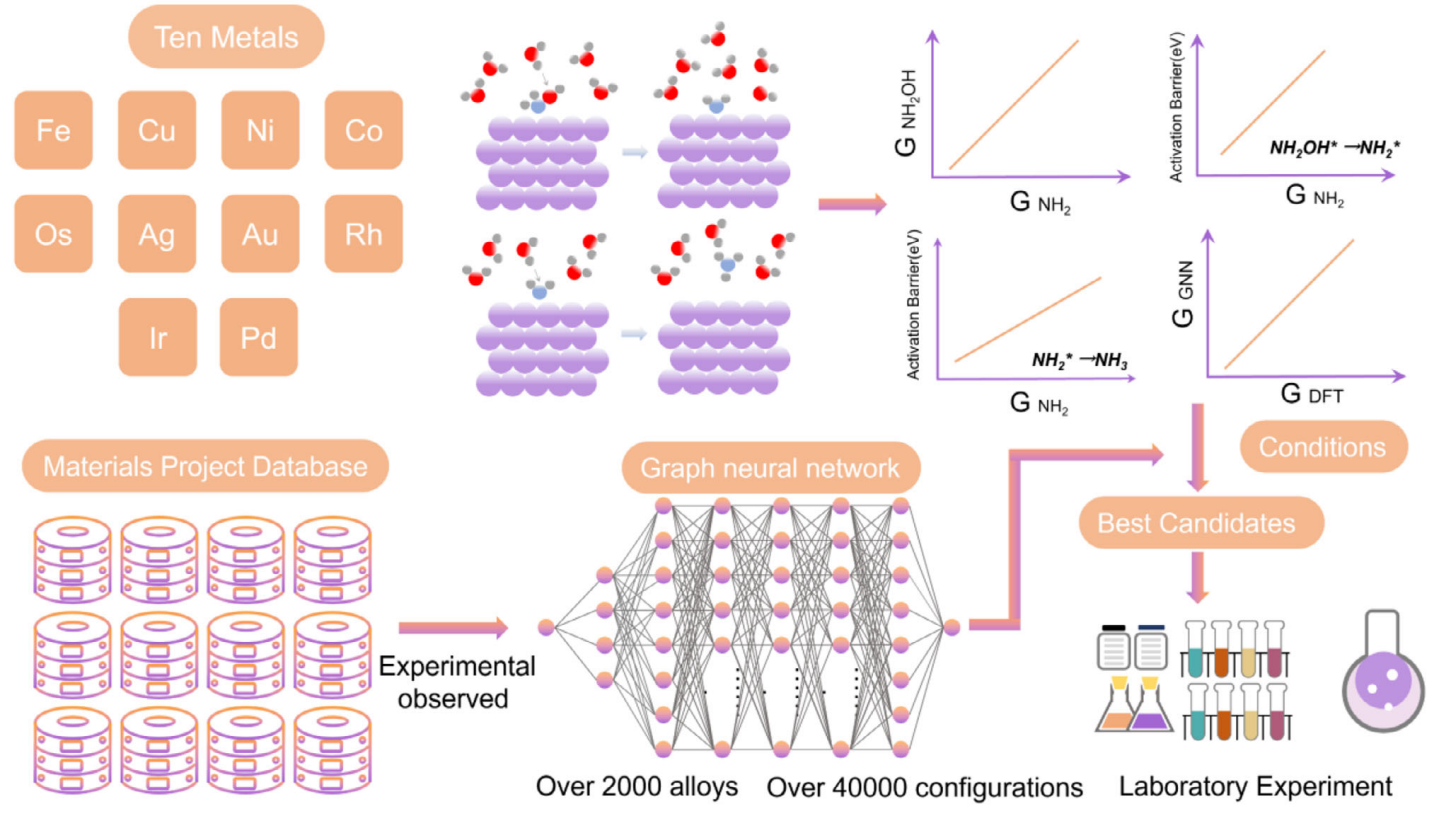
The activation energy barriers and the Gibbs energy changes of *NH_2_OH → *NH_2_+ OH^‐^ and *NH_2_+H_2_O → NH_3_+ OH^‐^+ * are obtained by DFT to construct the BEP relationships. The BEP relationships were employed with microkinetic theory to identify the optimal region with high activity and selectivity for ammonia production. Then, all experiment‐verified alloys in the Materials Project database were selected and optimized to obtain the most stable surface. GNNs were then applied to pre‐optimize and screen for the most promising candidates within the identified optimal region for further DFT calculations. Ultimately, we identified a system with optimal activity and stability for laboratory experiment validation.

A clear linear Brønsted–Evans–Polanyi (BEP) relationship was identified for each step in the hydrogenation of *NH_2_OH, indicating a predictable correlation between reaction barriers and Gibbs free energy changes, crucial for efficient catalyst design. Our calculations further revealed a strong linear relationship between the adsorption energies of *NH_2_ and *NH_2_OH, suggesting that the adsorption of NH_2_OH (the initial electroreduction step) can be effectively described by the *NH_2_ adsorption energy. As the energies of water and ammonia are fixed, the Gibbs free energy changes (ΔG) for all subsequent hydrogenation steps depend only on the adsorption energies of *NH_2_ and *H. As a result, both the ΔG and the activation barriers of each step in the NH_2_OH‐to‐NH_3_ conversion can be expressed in terms of *NH_2_ and *H adsorption energies. This allows the overall catalytic activity and selectivity (relative to H_2_ evolution) to be mapped as functions of just these two descriptors. Using microkinetic modeling, we constructed 2D heatmaps showing the activity and selectivity for NH_3_ across varying intervals of NH_2_ and H adsorption energy, identifying the regions where activity and selectivity are optimized. With the aid of the GNN, several systems (Cu_3_Sn(mp‐838), Cu_5_Sn_4_ (mp‐568524), Cu_5_Sn_4__2 (mp‐845), Cu_9_In_4_(mp‐683917), Cu_10_In_7_ (mp‐646039), CuMnSb (mp‐5866), and CuZn (mp‐987)) are filtered to satisfy this criterion from all experiment‐validated alloys from the Materials Project. To further ensure stability and account for potential errors in the BEP relationships and linear correlations, we conducted additional calculations to validate the performance of the screened alloys. Among these, the CuMnSb alloy was identified and experimentally confirmed to exhibit excellent performance, achieving high activity and NH_3_ selectivity. Such materials filtering process are presented in **Figure** **1**.

The activation energy barrier determines the reaction rate, representing the highest energy barrier from reactants to products. However, accurately obtaining the activation energy barrier requires optimizing the energy and geometry of the system along the reaction pathways to determine the transition state, which can be time‐ and resource‐consuming. Considering the large number of potential catalysts in the Materials Project, it is impractical to enumerate all possible transition states for all alloys to obtain the activation energy barrier and then identify one with the highest production rate and selectivity. To address this challenge, the BEP relationship offers a more practical solution. Such a relationship describes a linear correlation between a specific reaction's activation energy barrier and ΔG. This relationship allows us to gain the kinetic perspective from the thermodynamics energy space, which is much easier to compute and measure than the transition state. By establishing the BEP relationship for each reaction step in NH_2_OH reduction, we can adopt the ΔG as a reliable descriptor for catalytic activity, bypassing the transition state calculations.

### 2.2 BEP Relationship in the Aqueous Environment

However, the previous investigations into the BEP relationship focus on thermo‐catalysis, where the environment of the intermediates is relatively uniform and well‐characterized.^[^ ^17^ ^]^ Its application in the aqueous environment, particularly in electrochemical reactions, remains less understood and is a topic of ongoing investigation. In this research, we adopted the single‐layer water model to examine the effectiveness of BEP in electrochemical environments.^[^ ^18^ ^]^

**Figure 2:**
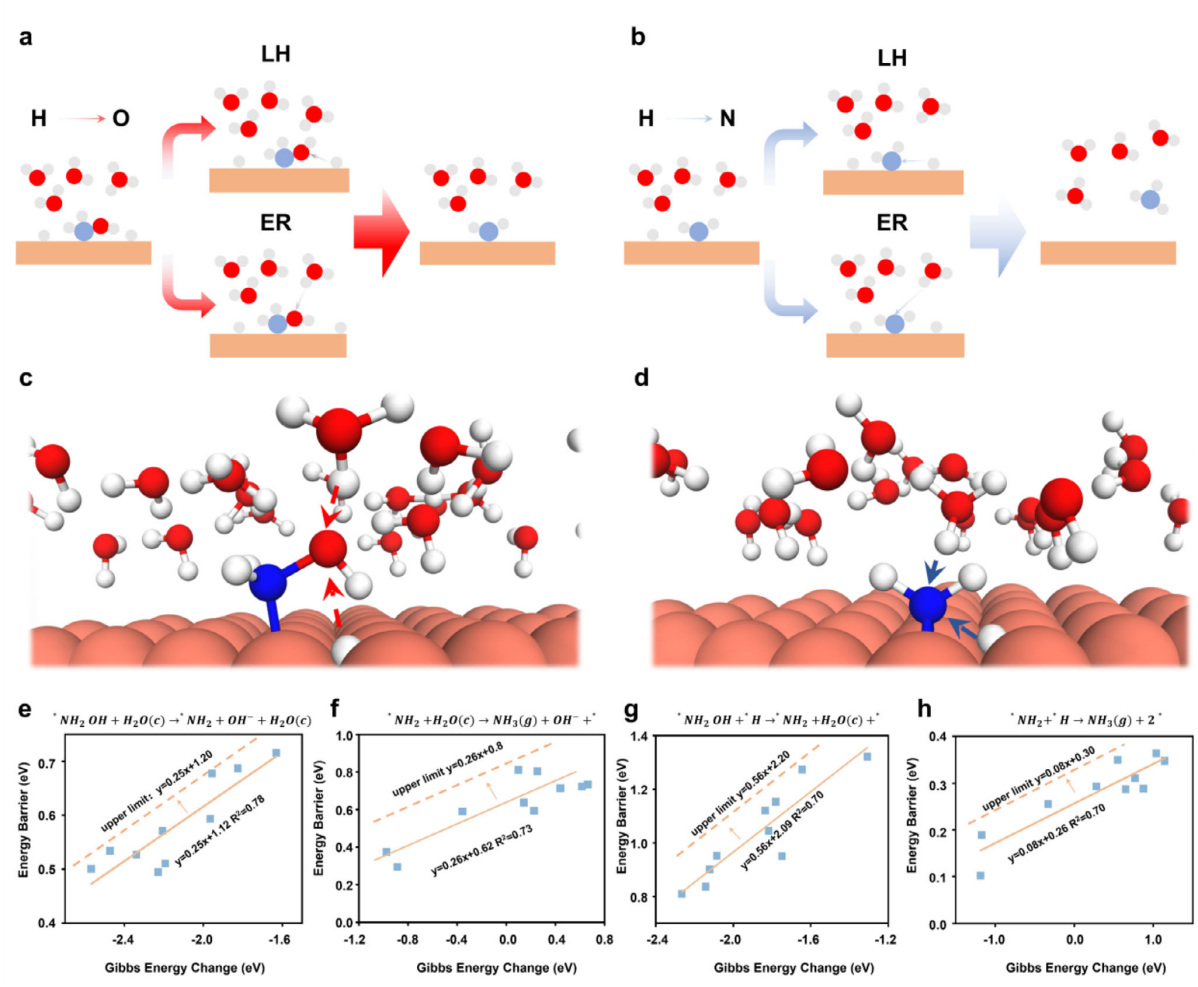
a,b) Sketch of the hydrogenation of *NH_2_OH to *NH_2_, then *NH_2_ to NH_3_ by ER and LH mechanisms. c,d) Structures of NH_2_OH and NH_2_ hydrogenation by ER and LH mechanisms on Cu. The BEP relationship of e)$*NH_{2}\mathrm{OH}\to *NH_{2}+OH^{-}$, f) $*NH_{2}+H_{2}O\left(c\right)\to NH_{3}\left(g\right)+OH^{-}+*$, g)$*NH_{2}\mathrm{OH}+*H\to *NH_{2}+*+H_{2}O\left(c\right),$ (h) $*NH_{2}+*H\to NH_{3}\left(g\right)+2*$.

As presented in **Figure** **2**a,b, we considered both ER and LH hydrogenation mechanisms to examine the existence of the BEP relationship under aqueous conditions. The hydrogenation of *NH_2_OH and *NH_2_ on a Cu slab was taken as a specific structural example, as illustrated in Figure 2c,d. The BEP relationships for various steps in the *NH_2_OH reduction process are presented in Figure 2e,h. These results demonstrate a pronounced linear correlation between the activation energy barrier and ΔG regardless of the hydrogenation mechanism and intermediate species involved. This confirms that the BEP relationship is preserved in the *NH_2_OH reduction process, even in the presence of a water layer. However, compared to the relatively stable thermal catalysis environment, introducing a water layer in electrocatalysis results in additional energy fluctuations (R^2^ lower than 0.8 in our BEP relationships). Such energy fluctuations caused by the water layer result in less precise linear correlations in the fitted BEP relationships. To consider the potential errors and computational inaccuracies, we adjusted the fitted BEP curves by increasing the intercept of the linear relationship while preserving the slope values. This adjustment effectively raises the perceived activation energy barrier for a given ΔG, providing a more stringent screening threshold. Adopting this stricter BEP model penalizes catalysts with a high activation energy barrier, even if their ΔG is favorable. These adjustments reduce the predicted activity of NH_2_OH reduction under working conditions and provide a more rigorous and reliable framework for screening materials. Consequently, promising catalysts identified through this method will likely demonstrate excellent performance in activity and selectivity. To account for possible fitting‐related uncertainties, we also introduced a lower‐bound BEP line by shifting the original BEP relationship downward, ensuring that all data points lie within an upper and lower limit, as shown in Figure S31 (Supporting Information). This approach offers a physically reasonable and bounded estimate of the uncertainty associated with BEP‐derived activation energies. The resulting uncertainty ranges—defined as the vertical distance between the upper and lower BEP lines—for the four key reaction steps are 0.11, 0.25, 0.22, and 0.10 eV, respectively. These values generally fall within the typical error margins of DFT calculations (≈0.2 eV), confirming that the BEP‐based predictions remain within a credible and reliable range.

### 2.3 Construction of Various Linear Relationships

**Figure 3:**
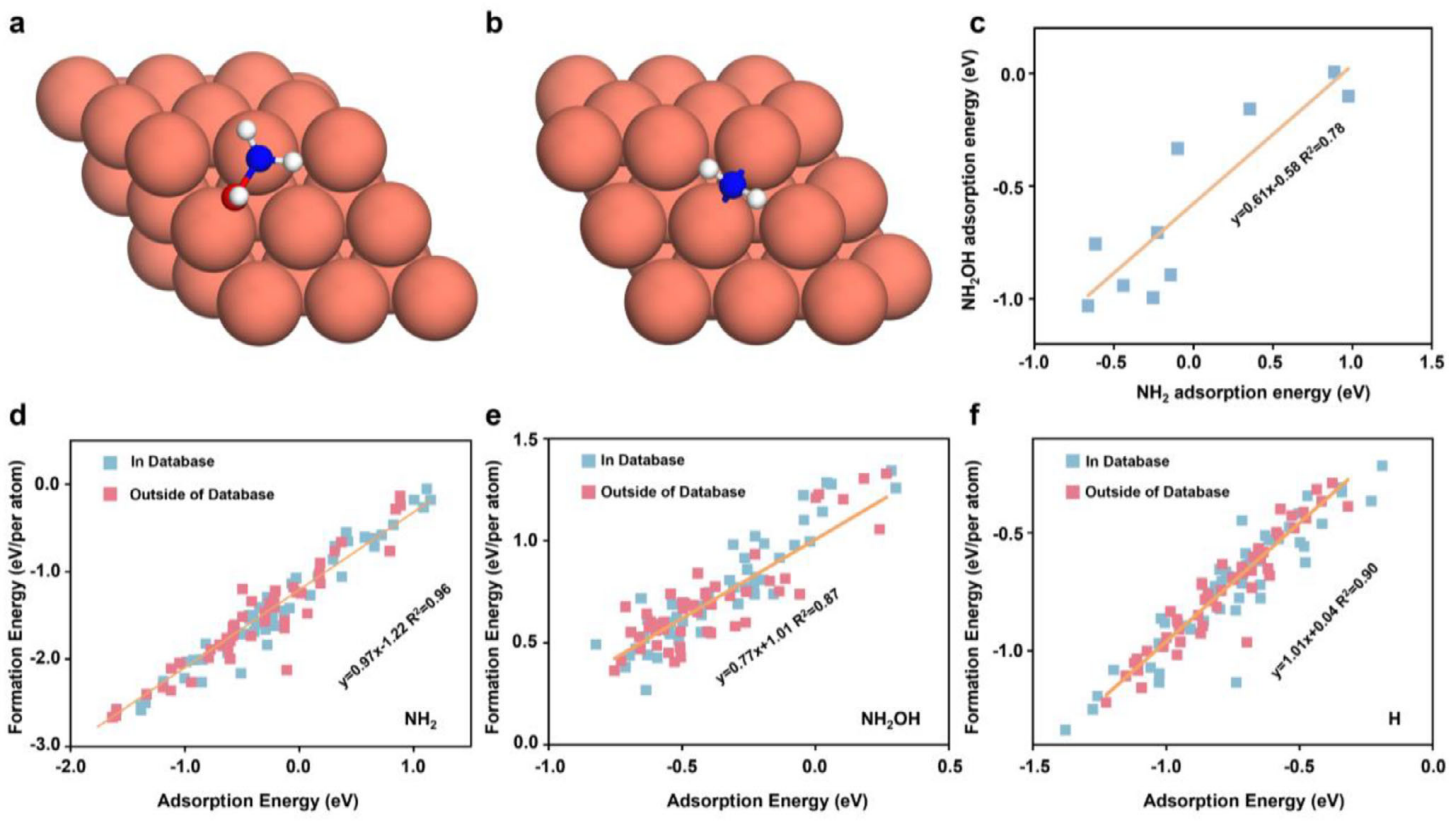
a,b) The optimized structure when NH_2_OH and NH_2_ are adsorbed on the Cu surface, individually. c)The linear relationship between the NH_2_OH adsorption energy and the NH_2_ adsorption energy. The linear relationship between the formation energy by GNN and the adsorption energy by DFT of d) NH_2_, e) NH_2_OH, and f) H. Both elements in and outside of the database were tested.

Although we established the BEP relationship, screening the extensive materials database in the Materials Project for suitable catalysts required the adsorption energies of NH_2_OH, ^*^NH_2_, and ^*^H on each material from DFT calculations. Such a challenging task requires us to reduce the reaction landscape's complexity further, making the subsequent screening process more efficient. Considering the typical adsorption modes of intermediates, ^*^NH_2_OH generally adsorbs in the on‐top site (**Figure** **3**a), while ^*^NH_2_ prefers a bridging configuration (Figure 3b), with the nitrogen atom serving as the adsorbing atom in both cases. We then explored the relationship between the adsorption energies of these two intermediates. A clear linear correlation was identified between the adsorption energies of ^*^NH_2_OH and ^*^NH_2_ in Figure 3c, enabling the adsorption energy of ^*^NH_2_OH to be expressed simply in terms of the adsorption energy of NH_2_. This significantly simplified the screening criteria by reducing the number of independent variables.

For the subsequent hydrogenation steps, the process primarily depends on the adsorption energies of ^*^H and ^*^NH_2_, with the energy of water and ammonia molecules remaining constant. The ΔG of each step in the NH_2_OH reduction pathway can be described by the adsorption energies of NH_2_ and H. Similarly, the activity of the HER is also determined by the adsorption energy of H. This relationship establishes that the reduction of NH_2_OH and its selectivity against HER for any given catalyst can be demonstrated by adsorption energies toward NH_2_ and H.

Despite the advantages of the descriptors derived from DFT calculations, conducting the adsorption energy calculations for all structures in the Materials Project by DFT remains a formidable challenge due to the immense computational resources required. To address this challenge, we turned to ML to accelerate the screening process. Among the diverse array of ML methods available, GNNs stand out for their ability to effectively model complex atomic systems. GNNs can dynamically learn representations from graph‐based data, where atoms are treated as nodes and chemical bonds as edges. It enables GNNs to capture both local and global structural features simultaneously. One of the most significant advantages of GNNs is their versatility in handling diverse chemical environments. Additionally, GNNs are inherently scalable, allowing them to rapidly process large datasets and predict properties like adsorption energies with a much lower computational cost than DFT calculations. This made GNNs indispensable for exploring vast chemical spaces efficiently, particularly suitable for our task.^[^ ^19^ ^]^ Given the varying accuracy and computational costs of different GNNs, we focused on EquiformerV2 (EqV2) for our model architecture, as it is currently the top‐performing model on several key leaderboards, including OC20,^[^ ^20^ ^]^ OC22,^[^ ^21^ ^]^ and ODAC23,^[^ ^22^ ^]^ surpassing numerous contributions from the broader scientific community. This model is designed to predict essential material properties, such as energy, forces, and stress, from an input structure. It is optimized for relaxed structure energy prediction, a critical task for materials discovery, by directly predicting forces and stress rather than relying on energy derivatives.^[^ ^23^ ^]^ Since the range of elements in the Materials Project might exceed those included in the EqV2 training set, we carefully compare the accuracy between the EqV2 and DFT for NH_2_, NH_2_OH, and H adsorption energy. Specifically, we selected 50 alloys from elements covered by the EqV2 training set and another 50 alloys containing elements not included in the EqV2 training set. As presented in Figure 2f,h, the correlation between GGN and DFT results is remarkably high regardless of the elements in the EqV2 training set, with R^2^ close to 0.9 and 0.96 for the H and NH_2_ adsorption energy. Such a strong correlation demonstrates that GNNs can be adopted as a surrogate model to predict a material's performance. These results can be projected into the DFT energy space through the above linear relationships for further analysis.

### 2.4 Microkinetic and DFT Screening

**Figure 4:**
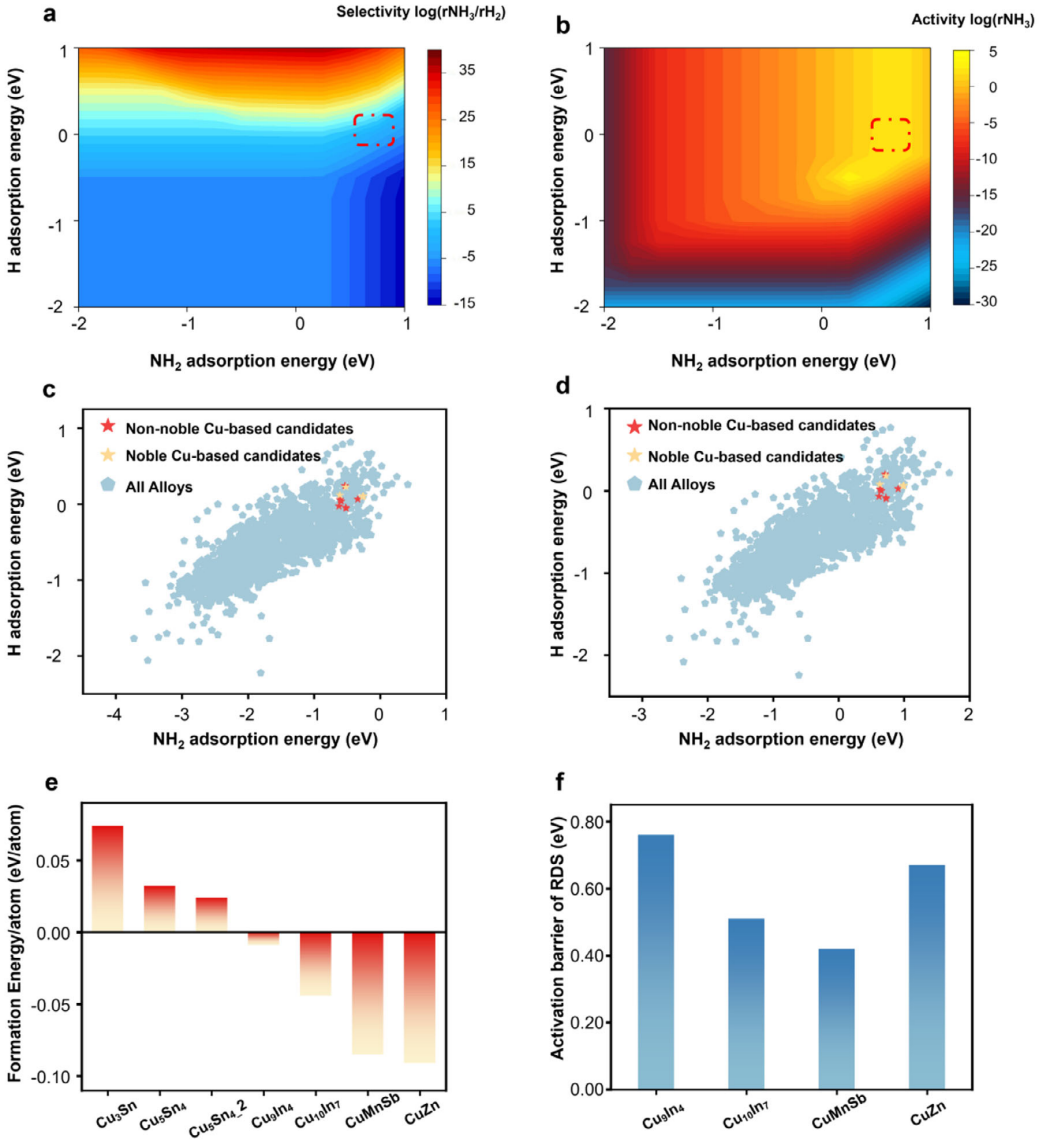
a) The selectivity of NH_3_ vs H_2_ and b) activity toward NH_3_ for different NH_2_ and H adsorption energy. The most promising candidates are in the highlighted areas of c) GNN and d) DFT. e) The stability and f) activity of non‐noble Cu‐based candidates based on the formation energy per atom and the RDS activation energy barrier.

Based on transition state theory and Arrhenius equations, the microkinetic modeling utilizes the reaction barrier and Gibbs energy change for each elementary step to calculate the overall reaction rate. Combined with steady‐state analysis, it provides a precise description of the overall reaction behavior. Results relating to selectivity and activity based on microkinetic modeling are summarized in **Figure** **4**a,b. These align well with our expectations and provide valuable insights into the total reaction pathways. A high NH_2_ adsorption energy results in strong NH_2_OH adsorption. This leads to surface poisoning of the metal catalyst, where the surface becomes saturated with *NH_2_OH, preventing further reaction.

On the other hand, when the adsorption energy toward NH_2_ is weak, it also weakens the adsorption energy toward NH_2_OH, making the NH_2_OH reduction difficult. Therefore, activity and selectivity are poor in the regions where the NH_2_ adsorption energy is either too strong or too weak. It is also important to note that even with the optimal NH_2_ adsorption energy range, it is crucial to consider the H adsorption energy, which significantly influences the competition between NH_2_OH reduction and the HER. If the metal surface strongly absorbs hydrogen, it can lead to surface poisoning due to excessive hydrogen coverage, limiting the adsorption sites available for NH_2_OH intermediates. Conversely, if the hydrogen adsorption is too weak, the surface hydrogenation of NH_2_ becomes challenging, reducing the overall activity of the NH_2_OH reduction process. Optimizing this delicate balance between NH_2_OH and hydrogen adsorption energy is critical for achieving high activity and selectivity. Thus, both H and NH_2_ adsorption energies must fall within an appropriate range to minimize the competing effects of surface poisoning and insufficient hydrogen. The red dashed rectangles in Figure 4a,b highlight the optimal area where activity and selectivity are promoted. This analysis also demonstrates the importance of tuning the adsorption energies to ensure the catalyst's efficiency in promoting NH_2_OH reduction over HER. We also constructed the microkinetic model based on the lower limit of the BEP relationship in Figure S32 (Supporting Information). The unchanged optimal area demonstrated the reliability of our simulation.

Next, we projected the energy space of all materials calculated by GNNs into the DFT energy space using the linear relationships shown in Figure 3d,f. This allowed us to efficiently predict the performance of a large set of alloy materials without performing exhaustive DFT calculations for each one. Combining these projections with the results of microkinetic modeling, we narrowed down the list of promising alloy candidates (Cu_3_Sn(mp‐838),

Cu_5_Sn_4_(mp‐568524), Cu_5_Sn_4__2(mp‐845), Cu_9_In_4_(mp‐683917), Cu_10_In_7_(mp‐646039), CuMnSb(mp‐5866), and CuZn (mp‐987)), as illustrated in Figure 4e. To conduct further screening, we first collected their formation energy per atom as a stability criterion from the Materials Project. We excluded the alloys with positive formation energy per atom, ensuring the materials selected for further analysis were thermodynamically stable (thus experimentally accessible). Considering the potential error in our BEP relationship, we performed additional DFT calculations on the remaining alloys to ascertain the activation energy barrier of the rate‐determining steps (Figure 4f). The detailed activation barriers of each step on these candidates are listed in Figure S27 (Supporting Information). We just present the barrier of the RDS here for clarity. This step helped us ensure our predictions were as accurate as possible. Notably, several alloy systems (Cu‐Zn,^[^ ^24^ ^]^ Cu‐Sn)^[^ ^5^, ^25^ ^]^ identified through our screening have recently exhibited excellent experimental performance, highlighting the robustness and reliability of our screening framework.

These comprehensive theoretical investigations identified the CuMnSb alloy as the most promising catalyst for NH_2_OH reduction to NH_3_. It shows favorable adsorption energies for NH_2_ and H, combined with good thermodynamic stability and optimal performance in microkinetic modeling, suggesting that it would offer excellent performance in NH_2_OH reduction. To validate this finding, we proceeded with experimental studies.

### 2.5 Experiment Validation

**Figure 5:**
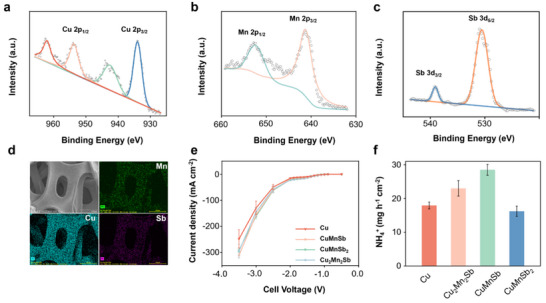
X‐ray photoelectron spectroscopy (XPS) data of a) Cu 2p, b) Mn 2p, and c) Sb 3d region for as‐prepared CuMnSb. d) Scanning electron microscopy (SEM) and elemental mapping images of as‐prepared CuMnSb. e) JV curves for different catalysts. f) NH_4_^+^ yield rate for different catalysts.

CuMnSb alloys were grown on Cu foam via a multi‐channel magnetic filtering cathodic vacuum arc deposition (FCVAD) technique, which can precisely control the deposition ratio of different metals. By controlling the deposition ratio of Cu, Mn, and Sb elements, we successfully prepared CuMnSb, CuMnSb_2_, and Cu_2_Mn_2_Sb ternary alloys on Cu foam. X‐ray photoelectron spectroscopy (XPS) was applied to investigate the composition and electronic structure of the as‐prepared catalysts. The Cu 2p XPS spectrum (**Figure** **5**a) of CuMnSb showed two peaks with binding energies of 933.8 and 953.8 eV (2:1 area ratio), corresponding to the Cu 2p_3/2_ and Cu 2p_1/2_ signals, respectively, of a surface Cu^2+^ species. A second set of peaks at higher binding energy corresponds to Cu^2+^ “shake‐up” satellites. A 0.2 eV positive shift compared to Cu foam (Figure S1, Supporting Information) demonstrated that the surface electronic structure changed after alloying. The Mn 2p XPS spectrum of CuMnSb showed peaks at 641.4 and 652.4 eV (2:1 area ratio, Figure 5b), corresponding to the Mn 2p_3/2_ and Mn 2p_1/2_ signals, respectively. The Sb 3d_5/2_ spectrum overlapped with the O 1s spectrum (Figure 5c), with the binding energy of the Sb 3d_3/2_ peak at 539.2 eV suggesting the presence of Sb^3+^ (as in Sb_2_O_3_). The XPS data, which probes the top few nanometers in the samples, thus verified the presence of oxidized states of Cu, Mn, and Sb in the near‐surface region in the as‐prepared CuMnSb catalyst (note: these oxidized species likely resulted from air‐exposure and would be reduced to the metallic state under the conditions of our electrocatalytic tests). Next, scanning electron microscopy (SEM) and energy‐dispersive spectroscopy (EDS) images were collected to characterize and confirm the homogeneous sputter of CuMnSb ternary alloy, as shown in Figure 5d. The prepared CuMnSb catalyst exhibited a similar surface morphology to the Cu foam, suggesting a thin CuMnSb coating of uniform thickness.

Our continuous plasma‐electrochemical system and membrane electrode assembly (MEA) were identical to those described in our recently published work.^[^ ^7^ ^]^ First, humidified air was purged into the plasma reactor, which was activated and converted into NO_x_H_y_ intermediates. These NO_x_H_y_ intermediates were then captured by a 1 M KOH solution, which was directly applied as the catholyte, whilst a 1 M KOH solution was used as the anolyte. All voltages reported in this study are full‐cell voltages without iR‐correction. The j–V curves for the different alloy catalysts are shown in Figure 5e. Among all tested catalysts, the CuMnSb ternary alloy catalyst exhibited the highest current density of 311.24 mA cm^−2^ at −3.5 V, followed by Cu_2_Mn_2_Sb, CuMnSb_2_, and the plain Cu foam. Although the CuMnSb_2_ ternary alloy catalyst had a higher current density than Cu foam at −3.5 V, its NH_3_ yield rate was the lowest among all ternary alloy catalysts. Besides, CuMnSb also presents the highest selectivity toward ammonia, as shown in Figure S8 (Supporting Information). As shown in Figure 5f, the CuMnSb ternary alloy catalyst achieved the highest NH_3_ yield rate of 28.47 mg h^−1^ cm^−2^, validating our theoretical predictions and strongly supporting our theoretical screening framework.

## 3 Conclusion

Achieving ammonia production directly from air is of great economic and scientific significance, addressing global challenges in sustainable chemical production. Leveraging the key intermediates in ammonia production from air, NH_2_OH, we integrated the GNN and microkinetic theory to identify the optimal non‐noble catalytic system for converting the NH_2_OH toward NH_3._ Guided by theoretical calculations, a non‐noble CuMnSb catalyst delivered an ammonia production rate of 28.47 mg h^−1^ cm^−2^ directly from air. This catalyst system significantly reduced our plasma‐electrochemistry device cost and provided a versatile framework for combining machine learning with advanced chemical technologies to accelerate catalyst design. While our work focuses on a relatively simple NH_2_OH‐to‐NH_3_ system, the machine learning–guided microkinetic screening framework we propose holds potential for broader applications. We acknowledge that the current model involves several simplifications—such as idealized surface structures, PCET assumptions, and limited solvent effects—which may limit its direct transferability to more complex systems. Nonetheless, this study establishes a foundational paradigm that can be progressively refined and extended to diverse catalytic reactions in future work. By bridging the gap between theoretical concepts and practical applications, this work paves the way for developing more efficient and cost‐effective catalytic systems, advancing fundamental science, and industrial innovation.

## Conflict of Interest

The authors declare no conflict of interest.

## Author Contributions

C.Z., X.G., and Z.J. contributed equally to this work. Z.W. and Y.C.L. conceived the project. C.Z. performed the DFT calculations and microkinetic modeling. X.G. performed all the catalyst tests. Z.J. and C.Z. performed the graph theory neural network simulation. C.Z. and Z.L. synthesize the catalysts. C.Z., X.G., and Z.J. wrote the main manuscript, and all authors contributed to the manuscript editing.

## Supporting information



Supporting Information

## Data Availability

The data that support the findings of this study are available from the corresponding author upon reasonable request.

## References

[1] M. Wang , S. Liu , T. Qian , J. Liu , J. Zhou , H. Ji , J. Xiong , J. Zhong , C. Yan , Nat. Commun. 2019, 10, 341. 30664636 10.1038/s41467-018-08120-x PMC6341113

[2] J. Deng , J. A. Iñiguez , C. Liu , Joule 2018, 2, 846.

[3] Z.‐H. Xue , S.‐N. Zhang , Y.‐X. Lin , H. Su , G.‐Y. Zhai , J.‐T. Han , Q.‐Y. Yu , X.‐H. Li , M. Antonietti , J.‐S. Chen , J. Am. Chem. Soc. 2019, 141, 14976. 31523954 10.1021/jacs.9b07963

[4] a) R. Tort , O. Westhead , M. Spry , B. J. V. Davies , M. P. Ryan , M.‐M. Titirici , I. E. L. Stephens , ACS Energy Lett. 2023, 8, 1003; 36816775 10.1021/acsenergylett.2c02697 PMC9926486

[5] a) J. Shao , H. Jing , P. Wei , X. Fu , L. Pang , Y. Song , K. Ye , M. Li , L. Jiang , J. Ma , R. Li , R. Si , Z. Peng , G. Wang , J. Xiao , Nat. Energy 2023, 8, 1273;

[6] a) S.‐L. Meng , C. Zhang , C. Ye , J.‐H. Li , S. Zhou , L. Zhu , X.‐B. Li , C.‐H. Tung , L.‐Z. Wu , Energy Environ. Sci. 2023, 16, 1590;

[7] X. Ge , C. Zhang , M. Janpandit , S. Prakash , P. Gogoi , D. Zhang , T. R. Cook , G. I. N. Waterhouse , L. Yin , Z. Wang , Y. C. Li , J. Am. Chem. Soc. 2024, 146, 35305. 39668149 10.1021/jacs.4c12858

[8] a) A. Zakutayev , N. Wunder , M. Schwarting , J. D. Perkins , R. White , K. Munch , W. Tumas , C. Phillips , Sci. Data 2018, 5, 180053; 29611842 10.1038/sdata.2018.53 PMC5881410

[9] a) L. Shen , J. Zhou , T. Yang , M. Yang , Y. P. Feng , Acc. Mater. Res. 2022, 3, 572;

[10] a) S. P. Ong , Comput. Mater. Sci. 2019, 161, 143; 10.1016/j.commatsci.2019.02.006 PMC7066999 32165790

[11] a) T. Xie , J. C. Grossman , Phys. Rev. Lett. 2018, 120, 145301; 29694125 10.1103/PhysRevLett.120.145301

[12] M. G. Evans , M. Polanyi , Trans. Faraday Soc. 1938, 34, 11.

[13] W. Xie , J. Xu , J. Chen , H. Wang , P. Hu , Acc. Chem. Res. 2022, 55, 1237. 35442027 10.1021/acs.accounts.2c00058 PMC9069691

[14] A. Jain , S. P. Ong , G. Hautier , W. Chen , W. D. Richards , S. Dacek , S. Cholia , D. Gunter , D. Skinner , G. Ceder , K. A. Persson , APL Mater. 2013, 1, 011002.

[15] J. Greeley , T. F. Jaramillo , J. Bonde , I. B. Chorkendorff , J. K. Norskov , Nat. Mater. 2006, 5, 909. 17041585 10.1038/nmat1752

[16] a) G. Kastlunger , P. Lindgren , A. A. Peterson , J. Phys. Chem. C. 2018, 122, 12771;

[17] a) A. Michaelides , Z. P. Liu , C. J. Zhang , A. Alavi , D. A. King , P. Hu , J. Am. Chem. Soc. 2003, 125, 3704; 12656593 10.1021/ja027366r

[18] I. Ledezma‐Yanez , W. D. Z. Wallace , P. Sebastián‐Pascual , V. Climent , J. M. Feliu , M. T. M. Koper , Nat. Energy 2017, 2, 17031.

[19] Z. Jiao , Y. Liu , Z. Wang , J. Chem. Phys. 2024, 161, 171001. 39484893 10.1063/5.0227821

[20] a) L. Chanussot , A. Das , S. Goyal , T. Lavril , M. Shuaibi , M. Riviere , K. Tran , J. Heras‐Domingo , C. Ho , W. Hu , A. Palizhati , A. Sriram , B. Wood , J. Yoon , D. Parikh , C. L. Zitnick , Z. Ulissi , ACS Catal. 2021, 11, 6059;

[21] R. Tran , J. Lan , M. Shuaibi , B. M. Wood , S. Goyal , A. Das , J. Heras‐Domingo , A. Kolluru , A. Rizvi , N. Shoghi , A. Sriram , F. Therrien , J. Abed , O. Voznyy , E. H. Sargent , Z. Ulissi , C. L. Zitnick , ACS Catal. 2023, 13, 3066.

[22] A. Sriram , S. Choi , X. Yu , L. M. Brabson , A. Das , Z. Ulissi , M. Uyttendaele , A. J. Medford , D. S. Sholl , ACS Cent. Sci. 2024, 10, 923. 38799660 10.1021/acscentsci.3c01629 PMC11117325

[23] a) Y.‐L. Liao , B. Wood , A. Das , T. Smidt , arXiv, 2023, arXiv:2306.12059;

[24] a) J. Lan , Z. Wang , C. W. Kao , Y. R. Lu , F. Xie , Y. Tan , Nat. Commun. 2024, 15, 10173; 39580449 10.1038/s41467-024-53897-9 PMC11585598

[25] a) Z. Xiang , Y. R. Lu , L. Meng , J. Lan , F. Xie , S. Gao , J. Li , M. Luo , M. Peng , Y. Tan , Adv. Mater. 2025, 37, 2501886; 10.1002/adma.202501886 40326147

