# Supplementary material for: Graph Neural Network Driven Exploration of Non‐Precious Metal Catalysts for Air‐to‐Ammonia Conversion
Source: Adv Mater. 2025 Aug 1;37(42):e09915. doi: 10.1002/adma.202509915 (PMC12548500; doi:10.1002/adma.202509915)
Supplement: Supplementary file 1 — Supporting Information [file ADMA-37-e09915-s001.pdf]

ADVANCED MATERIALS

Supporting Information

for *Adv. Mater.*, DOI 10.1002/adma.202509915

Graph Neural Network Driven Exploration of Non-Precious Metal Catalysts for Air-to-Ammonia Conversion

Chengyi Zhang, Xiaoli Ge, Zihao Jiao, Mengyao Chang, Chuang Zhao, Qingsong Hua, Zhaoqiang Li, Geoffrey I.N. Waterhouse, Yuguang C. Li* and Ziyun Wang**

Supporting Information

Graph Neural Network Driven Exploration of Non-Precious Metal Catalysts for Air-to-Ammonia Conversion

Chengyi Zhang,[†A] Xiaoli Ge,[†B] Zihao Jiao,[†A] Mengyao Chang,[A] Chuang Zhao,[C] Qingsong Hua,[C] Zhaoqiang Li,[*C] Geoffrey I.N. Waterhouse,[A] Yuguang C. Li,[*B] Ziyun Wang[*A]

A. School of Chemical Sciences, University of Auckland, Auckland, New Zealand

B. Department of Chemistry, University at Buffalo, State University of New York, Buffalo, New York, 14260, USA

C. Department of Physics, Faculty of Arts and Sciences, Beijing Normal University, Zhuhai, 519087, P.R. China

[†] These authors contributed equally to this work

*Corresponding author: lizq@bnu.edu.cn; yuguangl@buffalo.edu; ziyun.wang@auckland.ac.nz.

SUPPORTING INFORMATION

Contents

1. Theoretical Section 2
   1.1 Density Functional Theory Calculations 2
   1.2 Graph Neural Network Methods 4
   1.3 Gibbs energy and energy barrier calculations 5
   1.4 Microkinetic modeling 6
   1.5 Sensitivity Analysis of our microkinetic modeling 7
2. Experimental Section 9
   2.1 Catalysts Preparation 9
   2.2 Materials Characterization 9
   2.3 Electrochemical Experiments 10
   2.4 Product Quantification Methodology 10
   2.5 Plasma operation condition 11
3. Supporting Tables and Figures 12
   3.1 Reaction Pathways 12
   3.2 Materials Characterization 13
   3.3 Plasma-electrochemistry device 18
   3.4 Calculation Structures 21
   3.5 Microkinetic flow chart under different NH_2 and H adsorption energy 32
4. Supporting References 46

SUPPORTING INFORMATION

1. Theoretical Section

1.1 Density Functional Theory Calculations

All density functional theory (DFT) calculations were performed using the Vienna Ab-initio Simulation Package (VASP).[1] The plane-wave basis set was employed with a kinetic energy cutoff of 450 eV, ensuring convergence of total energies. The exchange-correlation interactions were described using the Perdew–Burke–Ernzerhof (PBE) functional within the generalized gradient approximation (GGA).[2] All symmetry operations were explicitly turned off to allow full structural relaxation without constraints. For the electronic smearing, Methfessel-Paxton first-order smearing was adopted with a width of 0.2 eV, suitable for metallic systems. The electronic self-consistency loop was considered converged when the total energy change between iterations fell below 10^{-7} eV, with at least 5 and at most 60 SCF iterations performed per ionic step. The electronic minimization was handled using the fast Davidson iteration scheme, and real-space projection operators were automatically selected to improve computational efficiency. Medium precision was used for the FFT grid, and Grimme's DFT-D3 dispersion corrections were included to account for van der Waals interactions.[3] The Brillouin zone was sampled using a Γ-centered Monkhorst-Pack grid of 3 × 3 × 1, which was sufficient to converge the total energy for the chosen surface model.[4] Our calculations have taken the spin-polarized effect into account. Ni, Co, and Fe magma were tested individually in primitive cells to obtain a stable structure. All the adsorption geometries were optimized using a force-based conjugate gradient algorithm, while transition states (TSs) were located with a constrained minimization technique[5]. To mimic realistic surface conditions while minimizing computational cost, the bottom two atomic layers were fixed, and only the top two layers were allowed to relax. This partial relaxation approach is commonly employed in surface calculations to reduce spurious slab polarization and simulate the semi-infinite bulk beneath the surface. The vacuum layer was set sufficiently large (typically >15 Å) to prevent spurious

interactions along the normal surface between periodic images. Initial charge densities were generated by atomic superposition.

Geometry optimizations were performed using a conjugated gradient algorithm with a time step of 0.2 fs. The force convergence criterion was set to 0.05 eV/Å. The stress tensor was computed, but only atomic positions (not cell shape or volume) were allowed to relax. For the geometry optimization, implicit solvation effects were included using the VASPSOL model, with a surface tension-related cavity energy term set to 80 to express the aqueous environment.[6] For the explicit solvation structure, water densities were chosen to be close to that of the Pt(111) bilayer structure found in UHV experiments within the unit cell sizes considered.[7] Water layer structures were determined using a minima-hopping algorithm that alternates between Molecular Dynamics and geometry optimization steps to construct a series of local minima.[8] To simulate a charged double layer at the electrochemical interface, a single hydrogen atom was placed in the water layer. The ground-state electronic structure redistributes the charge from this atom's one electron to the metal, creating a charge-separated double layer.[9] The GC-DFT calculations were performed in the JDFTx, we adopted the optimized structure from the VASP package and calculated the single-point energy under different potentials to test the reliability of our solvation model. The Brillouin zone was sampled using a uniform 3×3×1 Gamma-centered k-point mesh, suitable for surface slab geometries. Ultrasoft pseudopotentials from the GBRV library were employed for all elements. The Perdew-Burke-Ernzerhof (PBE) generalized gradient approximation was used for exchange-correlation interactions. A plane-wave kinetic energy cutoff of 20 Hartrees was applied. Electronic minimization was performed with a convergence threshold of 1×10^{-5} Hartree and up to 1000 iterations. Finite-temperature Fermi smearing of 0.1 eV was used. Implicit solvation was modeled using the CANDLE variant of the LinearPCM method with water as the solvent, and ionic concentrations of 0.1 M K^+ and F^- were included. A target

electron chemical potential was set and van der Waals interactions were included. All calculations were spin-polarized along the z-axis.

1.2 Graph Neural Network Methods

We obtained all bulk structures (over 2 thousand) composed of p-group metals and transition metals from the Materials Project (excluding lanthanide series, radioactive elements, and toxic elements such as Hg, Tl, and Pb). For each bulk structure, the crystal plane corresponding to the strongest peak in its XRD spectrum was considered as the most stable plane. The slabs with different terminations were generated using Pymatgen.[10] We choose the termination with the smallest surface atomic height variance, as this can make the coordination number of surface atoms as large as possible without the appearance of isolated atoms on the surface, which means it may be more stable under experimental conditions The adsorbate intermediates including *H, *NH_2 and *NH_2OH with the N atom as binding sites were placed on 10 random adsorption sites in each slab and about 40 thousand adsorption configurations were generated.

Directly using DFT to perform geometric optimization of all adsorption configurations is computationally time-consuming. The OpenCatalystProject (OCP) provides multiple pretrained machine learning potentials (MLP) trained over 1.2 million DFT relaxations.[11] The **EquiformerV2** is the current state of the art among them,[12] according to the OCP leaderboard, reaching an average of 0.227 eV energy MAE and 0.013 eV/A forces MAE over the extensive element composition spaces. For each intermediate type, 100 samples were chosen to run geometric optimization using EquiformerV2 MLP and DFT calculations. Structure optimization uses the BFGS algorithm implemented within ASE, with force convergence of 0.05 eV $Å^{-1}$. The high Pearson correlation coefficients indicate that the energy predicted by EquiformerV2 is consistent with the DFT calculation. The EquiformerV2 MLP was used as the surrogate model to run optimization, and the lowest adsorption energy in each slab was selected for the catalysts screening.

1.3 Gibbs energy and energy barrier calculations

We utilized the computational hydrogen electrode model to calculate reaction energies as a function of potential. At a potential of U=0 V versus RHE, protons and electrons are in equilibrium with hydrogen gas (H_2) under standard conditions applicable across all pH levels:[13]

$$\mathrm{H^+ + e^- \rightarrow H_{2(g)}}$$

At a given U≠ 0V versus RHE,

$$\mu(\mathrm{H^+}) + \mu(\mathrm{e^-}) = \frac{1}{2}\mu(\mathrm{H_2}) - \mathrm{eU}$$

for calculating the Gibbs energy change of the energy barrier. To evaluate the effect of applied potential on proton-coupled electron transfer (PCET) steps, we employed a symmetry factor (β) of 0.5.[14] This value reflects the assumption that the transition state involves the transfer of half an electron, which is commonly adopted in electrochemical kinetics when the charge transfer is concerted but the exact charge distribution at the transition state is unknown. Accordingly, the potential-dependent correction to the reaction barrier ($\Delta G^{\ddagger}(U)$) was introduced as:

$$\Delta G^{\ddagger}(U) = \Delta G^{\ddagger}(0) - \beta e U$$

where $\Delta G^{\ddagger}(0)$ denotes the activation barrier at the reference potential (typically 0 V vs. RHE), e is the elementary charge, and U is the applied electrode potential. This correction effectively accounts for the electrostatic stabilization of the transition state in response to increasing overpotential. The use of β = 0.5 ensures a balanced treatment of the potential influence, consistent with prior theoretical treatments of PCET processes in electrocatalysis. In addition, in microkinetic modeling, we directly represent the influence of pH on the overall reaction kinetics through the concentration of proton donors or acceptors (e.g., H^+), which are modulated in accordance with the pH value. To further confirm the reliability of the CHE

model in our system, we adopted the hydrogenation of *HCCH on Cu on different potential vs. SHE in Figure S28. Our results confirm the linear change of the hydrogenation barrier with potential, indicating the reliability of our model.

1.4 Microkinetic modeling

In microkinetic modeling, the reaction network is constructed from a series of elementary steps, and fundamental thermodynamic and kinetic principles govern the net rate of each step. According to the De Donder relation, the net rate r_i for an elementary step i can be expressed as a product of the forward rate constant k_i. The surface coverages θ_j of the participating reactants, their stoichiometric coefficients v_{ij}, and a reversibility factor ($1-Z_i$), where Z_i represents the thermodynamic driving force for reversibility.[15] Mathematically, this is written as:

$$r_i = k_i \prod_j \theta(j)^{v_{ij}}(1 - Z_i)$$

Here, $Z_i = \prod_j \theta(j)^{v_{ij}}/K_{\mathrm{eq},i}$, where $K_{\mathrm{eq},i}$is the equilibrium constant of step i, given by

$$K_{\mathrm{eq},i} = \exp(-\Delta G_i/RT)$$

This term reflects the extent to which the step is reversible:$Z_i \to 0$ for irreversible steps and $Z_i \to 1$ for steps approaching equilibrium. The stoichiometric coefficient v_{ij} indicates the number of molecules of species j involved in step i.

To determine which elementary step most strongly influences the overall reaction rate, we employ the degree of rate control (DRC), a sensitivity metric introduced by Campbell and co-workers.[16] The DRC for a transition state i quantifies the relative change in the overall reaction rate r concerning a perturbation in the Gibbs free energy of that transition state, while holding the energies of all other species constant. It is formally defined as:

$$\mathrm{DRC}_i = \frac{\partial \ln r}{\partial\left(-G_i^{\ddagger}/RT\right)}$$

This metric captures how much the rate-limiting character of a particular transition state contributes to the net rate, allowing mechanistic insight beyond conventional energy barrier comparisons. A larger DRC value indicates greater control over the overall reaction rate by that particular transition state.

Similarly, the importance of the thermodynamic stability of an intermediate species n can be evaluated through a corresponding DRC definition:

$$\mathrm{DRC}_n = \frac{\partial \ln\, r}{\partial(-G_n/RT)}$$

This formulation identifies how changes in an intermediate's standard Gibbs free energy affect the overall reaction rate, assuming all other species remain unchanged. These DRC values provide a rigorous way to dissect the interplay between kinetics and thermodynamics across complex catalytic networks.

In terms of input conditions, the model operates under room temperature. The products were set to be 10^{-20} bars, to describe the reaction condition at the beginning. Mass transport limitations were not included in the modeling. The proton concentration was limited by setting the maximal proton concentration near the surface to 10^{-pH}.

1.5 Sensitivity Analysis of microkinetic modeling

When fitting the Brønsted–Evans–Polanyi (BEP) and linear-scaling relations, we must account for two sources of uncertainty: (i) solvent-phase effects, which introduce additional scatter in aqueous media, and (ii) the intrinsic 0.2 eV accuracy limit of standard GGA-DFT. To quantify how these errors might bias our predictions, we constructed an **uncertainty envelope** by rigidly translating the best-fit BEP line up and down by ΔE, generating an **upper-limit** and a **lower-limit** correlation that bracket every data point. Each bounding line was then used to re-parameterise the micro-kinetic model and to rescreen the entire catalyst set. The resulting optimal adsorption-energy window—and the corresponding shortlist of top catalysts—changed

only marginally between the upper- and lower-limit cases. This consistency indicates that our methodology is robust against typical solvent and DFT errors and underscores the reliability of the screening results.

2. Experimental Section

2.1 Catalysts Preparation

The CuMnSb ternary alloys on Cu foam were prepared through the multi-channel magnetic filtering cathodic vacuum arc deposition (FCVAD) technique (DCLD-600) under a negative bias voltage of 70 V with various arc currents. During this process, CuMn (50 at% Cu, 50 at% Mn, purity 99.9%), Cu (purity 99.9%) and Sb (purity 99.9%) were employed as target materials, Cu foam as substrate, and the deposition time was 2 minutes. For CuMnSb alloy, the arc current was set to be 65 A current for CuMn arc and 75 A current for Sb arc (abbreviated as 65 A-CuMn, 75 A-Sb). For the $CuMnSb_2$ and Cu_2Mn_2Sb alloys, the parameters were (60 A-CuMn, 80 A-Sb) and (70 A-CuMn, 70 A-Sb), respectively. FCVAD is a low-temperature and plasma-assisted deposition method that uses a magnetic field to remove macro-particles and droplets from the plasma stream, enabling the deposition of dense, uniform, and compositionally controlled films.[17] It is particularly effective for synthesizing amorphous or nanostructured multi-element alloys with high purity. The film thickness can be easily controlled by adjusting the deposition time. These advantages make FCVAD well-suited for preparing high-quality thin-film catalysts, such as the $CuMn_xSb_\gamma$ alloy in our study. Besides, it is difficult to measure the film thickness on the porous Cu foam substrate, the same deposition conditions were applied to a flat Si substrate for comparison. As shown in **Figure S10**, the thickness of the deposited film is approximately 75 nm. The corresponding deposition rate is 37.5 nm/min.

2.2 Materials Characterization

Scanning electron microscopy (SEM) and energy-dispersive X-ray spectroscopic (EDS) images were obtained on HITACHI, Regulus 8100, and Bruker XFlash 630M The surface compositions of the different ternary alloys were carried out using X-ray photoelectron

spectroscopy (XPS) (PHI 5000 Versaprobe). ^{1}H-NMR spectra were measured by the Bruker 500-MHz system.

2.3 Electrochemical Experiments

All electrochemical experiments were conducted at room temperature using a Squidstat Plus Potentiostat (Admiral Instrument). All electrochemical evolutions were performed in triplicate to get the error bars. The air flow rate was set to 400 sccm and controlled by a mass flow meter. For the MEA electrolyzer, the ternary alloy catalysts and Ni foam were used as the cathode and anode, separated by an anion exchange membrane (AEM). A 1 M KOH solution containing plasma-generated NO_xH_y intermediates was applied as the catholyte, while another 1 M KOH solution was used as the anolyte. The reaction area was 1 cm^2. The j-V curves of different ternary alloy catalysts were obtained through chronoamperometry at different voltages for 5 mins. The current densities were averaged over the final minute of the measurement.

2.4 Product Quantification Methodology

The concentration of NH_4^+ was determined by ^{1}H NMR spectroscopy. NMR samples were prepared by adding 600 μL of electrolyte collected after electroreduction and 60 μL of 9 M H_2SO_4 to the NMR tube. Then, a coaxial insert containing standardized DMSO in D_2O was used as the internal reference. The setup for the coaxial tube and calibration curve is illustrated in Figure. S14.

In our system, humid air is introduced into the plasma, resulting in the formation of a complex mixture of nitrogen-containing species, including various NO_x (e.g., NO, NO_2), and nitrogen–hydrogen–oxygen compounds such as NH_2OH and related intermediates. These species are generated simultaneously through non-thermal plasma processes and often undergo rapid interconversion. As a result, quantitatively measuring the concentration of each individual product is extremely challenging. Besides, according to our previous study in the same system, the average number of electron transfers of NH_4^+ production can be evaluated under the same

experimental conditions to estimate the NH_2OH ratio from the plasma system. The corresponding Faradaic efficiency (FE) of ammonia is 98.7%, calculated as FE (H_2) is 1.3%. Based on these values, we estimate the average number of electrons transferred per mole of NH_3 using the following equation:

$$n = \frac{FE * Q}{F * C * V * 100\%} = \frac{98.7\% * 360\ C}{96485 \frac{C}{mol} * \frac{40.9 \frac{mg}{h * cm^2} * 0.5\ h * 1\ cm^2}{18 \frac{g}{mol} * 1000 \frac{mg}{g} * 100\%}} = 3.25$$

For reference, the electron transfer numbers for the conversion of NH_2OH, NO_2^- , and NO_3^- to NH_3 are 2, 6, and 8, respectively. The observed electron transfer number of ~3.25 thus suggests that NH_2OH contributes substantially to the overall ammonia production. Moreover, the average electron transfer number of 3.25 highlights the importance of the final hydrogenation steps toward ammonia formation. Focusing on these steps could enhance the overall activity of the plasma–electrochemical system, which also justifies our emphasis on NH_2OH as a key intermediate.

2.5 Plasma operation condition

For the plasma operation condition, the voltage waveform of the AC power supply indicates a peak voltage of ~16 kV and a frequency of ~6 kHz. The power consumption of the plasma reactor was measured to be ~150 W using a wattmeter connected directly to the wall power supply, as presented in Figure S15.

3. Supporting Tables and Figures

3.1 Reaction Pathways

R1	NH_2OH+* ↔ *NH_2OH
R2	*NH_2OH ↔ *NH_2+OH(c)
R3	*NH_2+H_2O(c) ↔ NH_3(g)+OH(c)+*
R4	*NH_2OH+*H ↔ *NH_2+H_2O(c)+*
R5	*NH_2+*H ↔ NH_3(g)+2*
R6	H_2O(c)+ * ↔ *H+OH(c)
R7	*H+H_2O(c) ↔ H_2(g)+OH(c)+ *
R8	*H+*H ↔ H_2(g)+2*

Table. S1 The reaction pathway of the whole system. *denotes the adsorbate species, (g) denotes the gas phase, and (c) denotes the liquid state.

3.2 Materials Characterization

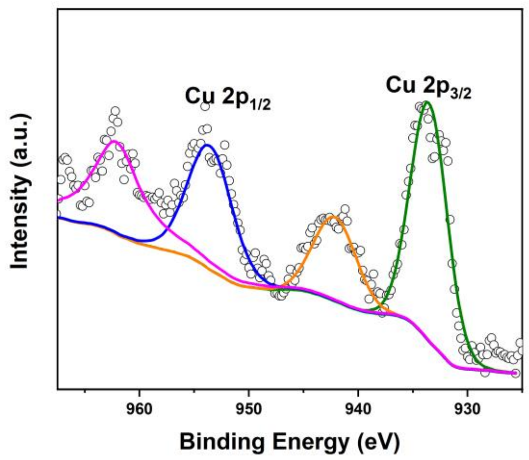

Figure S1. X-ray photoelectron spectroscopy (XPS) spectra of Cu 2p of Cu foam.

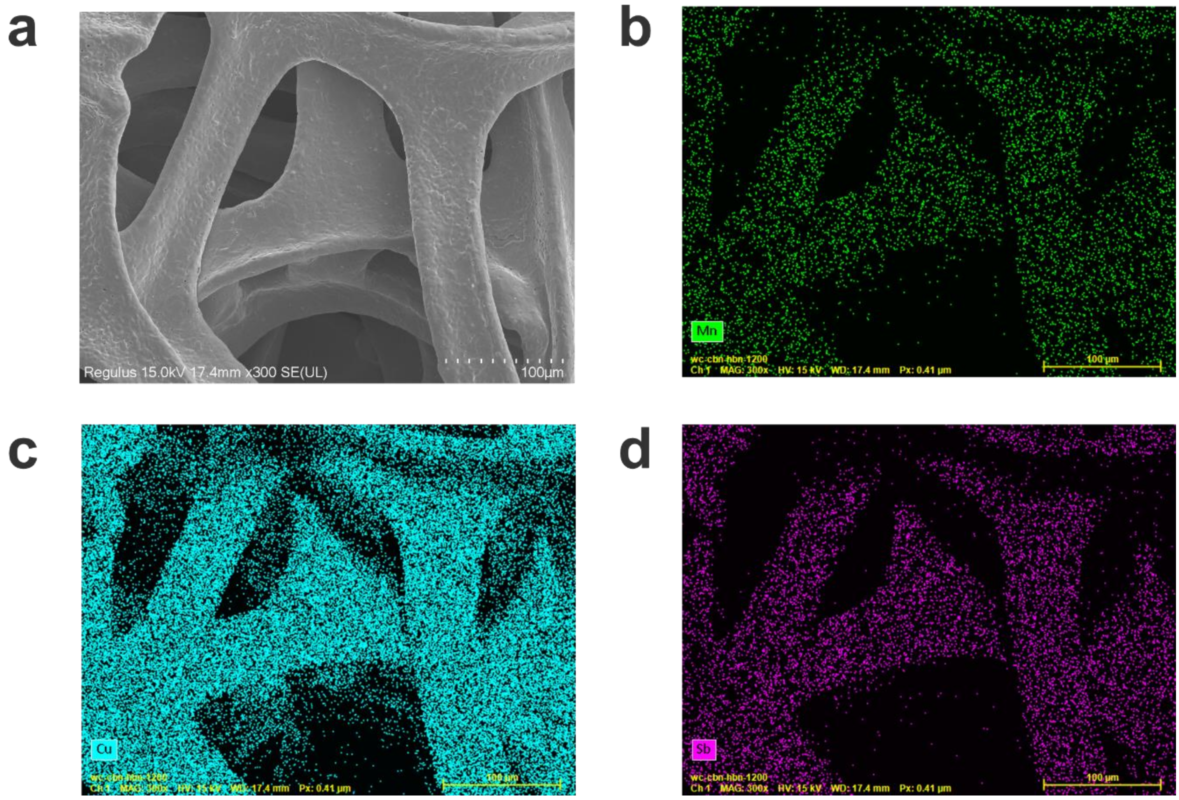

Figure S2. Scanning electron microscopy (SEM) and elemental mapping images of prepared $CuMnSb_2$.

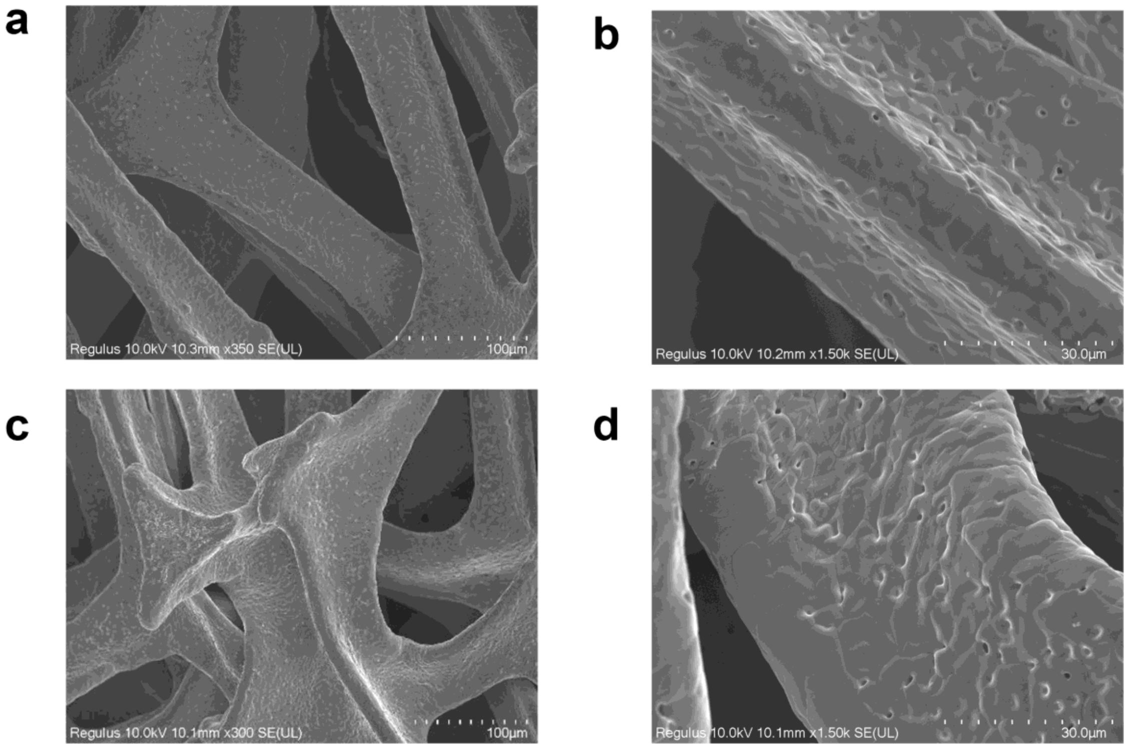

Figure S3. Scanning electron microscopy (SEM) images of the plain (a-b) Cu foam and (c-d) prepared Cu_2Mn_2Sb.

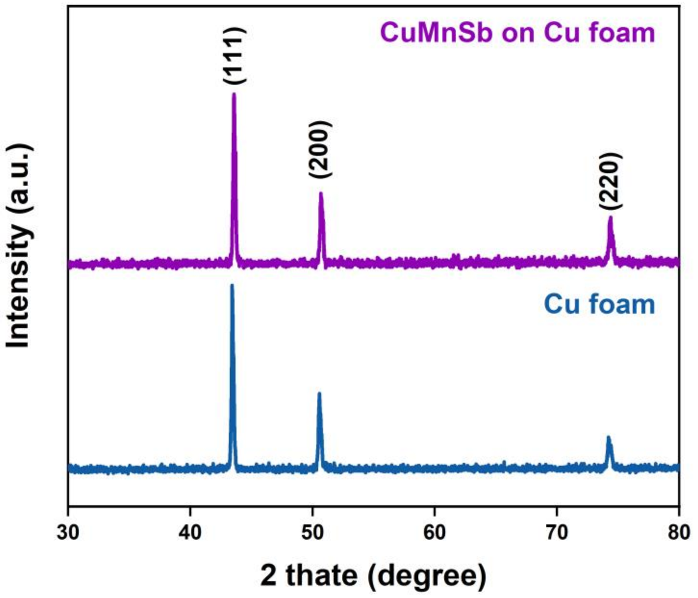

Figure S4. The XRD patterns of the synthesized CuMnSb catalyst deposited on Cu foam and pure Cu foam substrate.

SUPPORTING INFORMATION

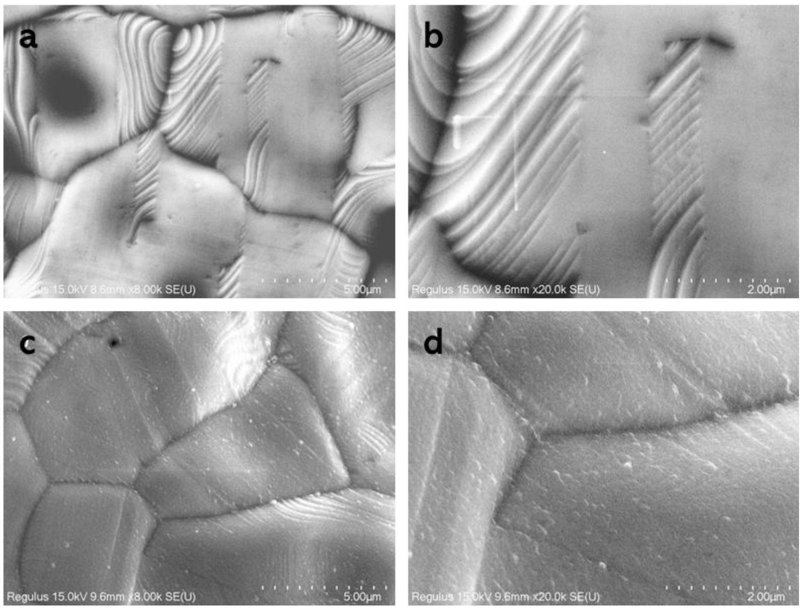

Figure S5. The high-resolution SEM images for the (a-b) pure Cu foam and (c-d) synthesized CuMnSb catalyst deposited on Cu foam substrate.

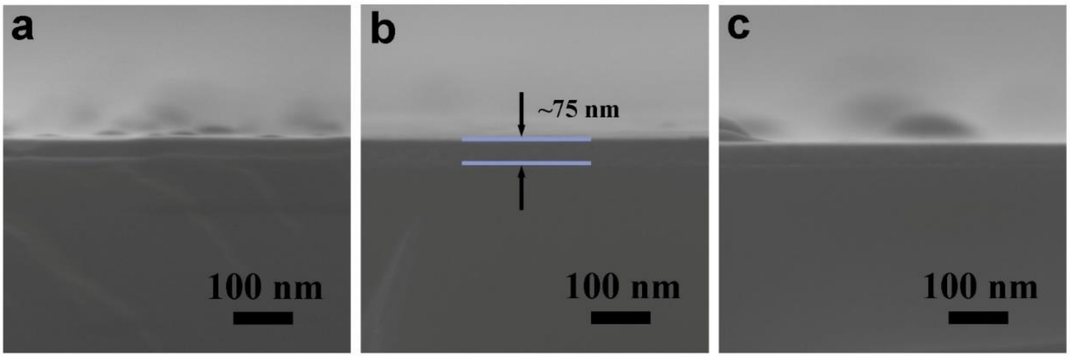

Figure S6. The cross-sectional SEM images for the synthesized (a) Cu_2Mn_2Sb, (b) CuMnSb, and (c) $CuMnSb_2$ alloys deposited on a Si substrate.

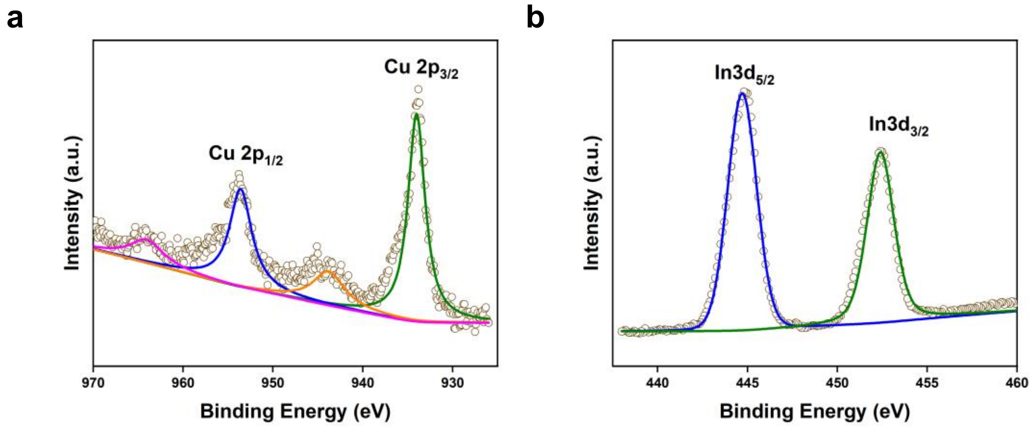

Figure S7. The XPS spectra of (a) Cu 2p, (b) In 3d for the prepared Cu-In alloy.

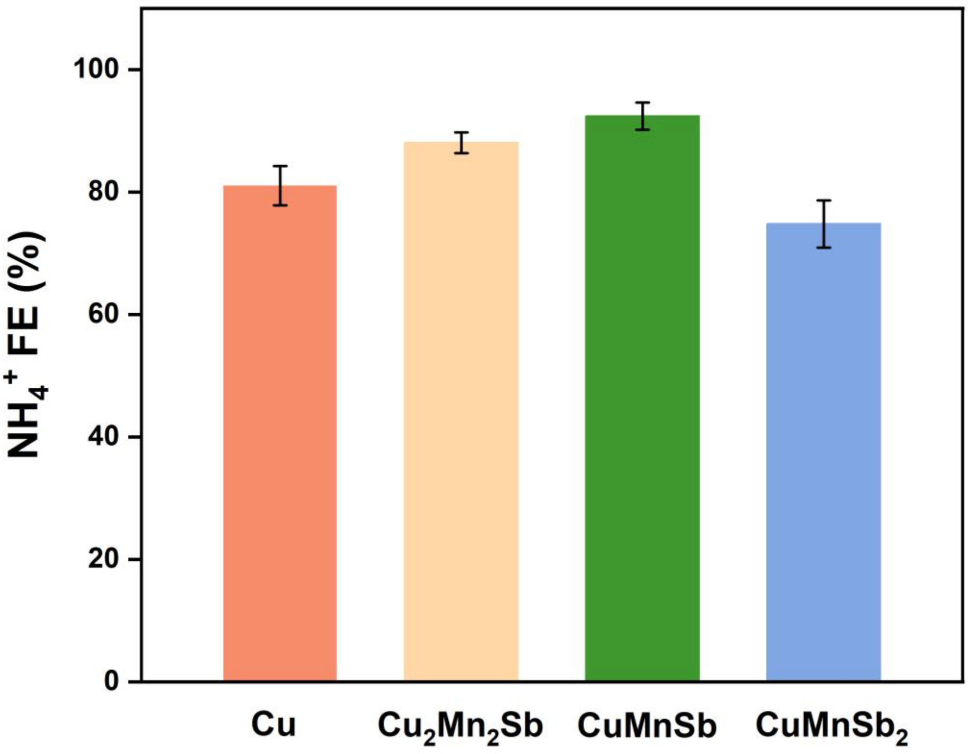

Figure S8. Comparison of the ammonia FE of different electrocatalysts.

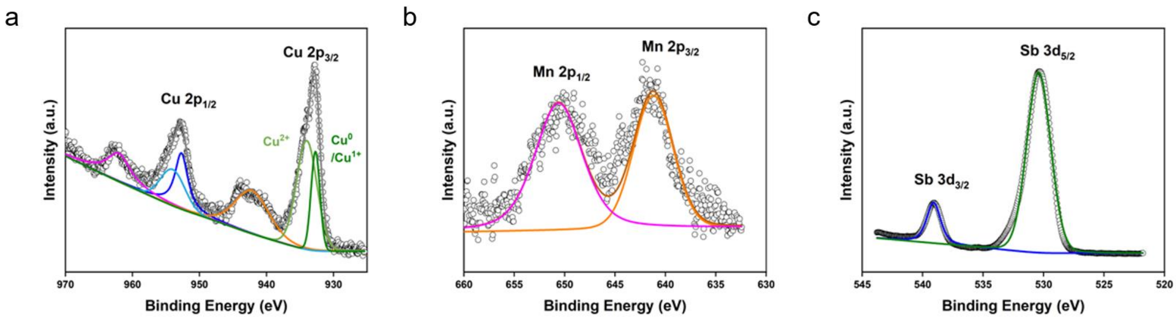

Figure S9. The XPS spectra of (a) Cu 2p, (b) Mn 2p, and (c) Sb 3d of CuMnSb catalyst after electrochemical reaction.

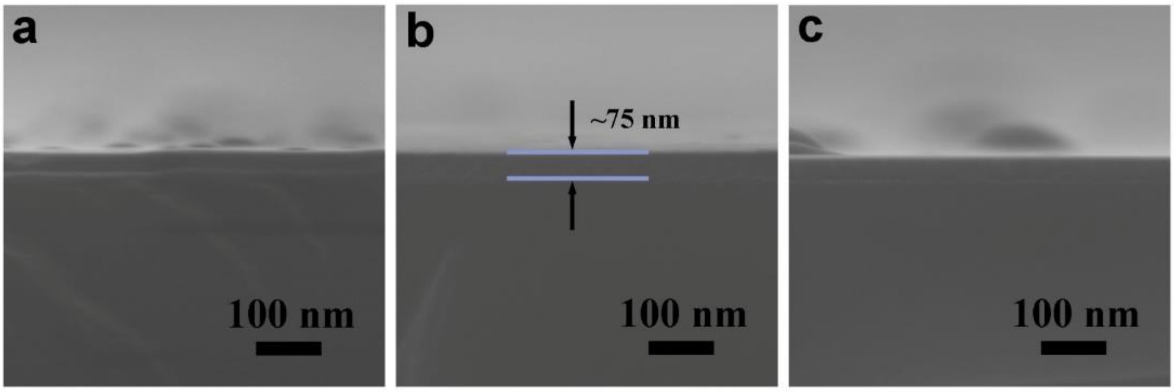

Figure S10. The cross-sectional SEM images for the synthesized (a) Cu_2Mn_2Sb, (b) Cu_2Mn_2Sb, and (c) $CuMnSb_2$ alloys deposited on a Si substrate.

3.3 Plasma-electrochemistry device

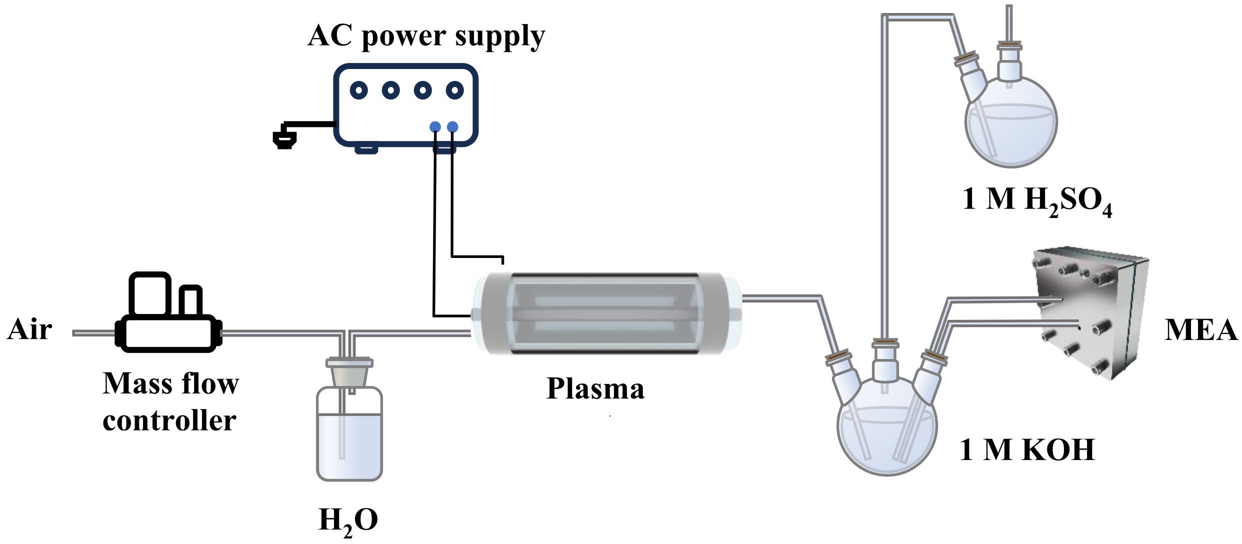

Figure S11. Schematic diagram of our proposed plasma electrocatalyst-integrated system for high NH_4^+ production rate.

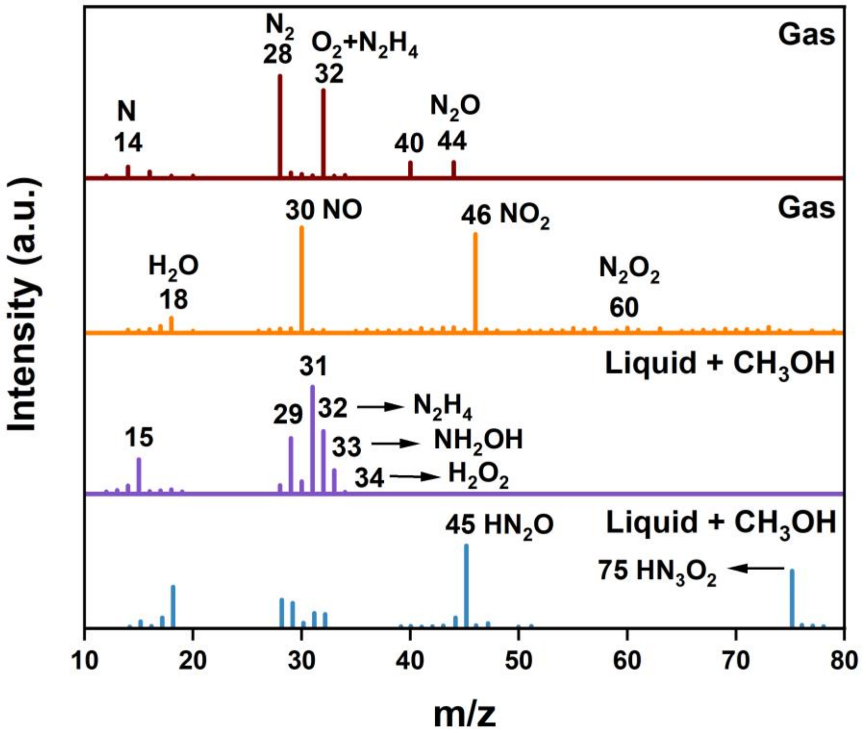

Figure S12. Mass spectra of the products generated by the plasma reactor with humidified air as the feed gas. Liquid-phase products were collected by bubbling them into methanol for GC-MS analysis. Orange and wine lines represent the gaseous products, while the purple and blue lines correspond to the liquid products.

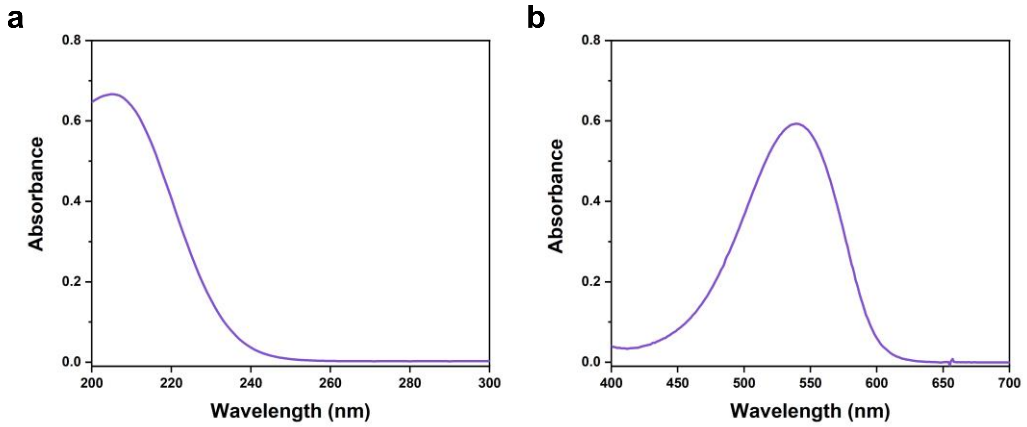

Figure S13. UV-vis absorption spectrum of our plasma-generated NO_3^- and NO_2^-.

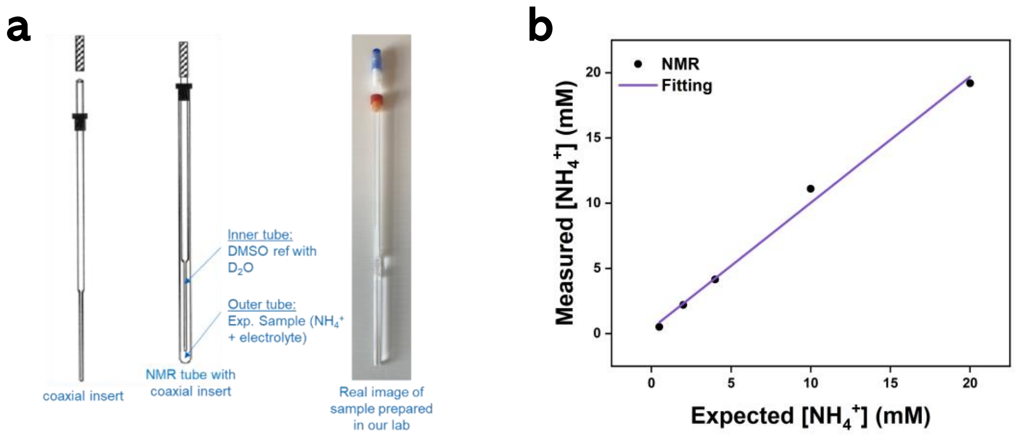

Figure S14. (a) The illustration and real image of the NMR coaxial insert tube. (b) Concentration of NH_4^+ determined by NMR for standard NH_4^+ solutions.

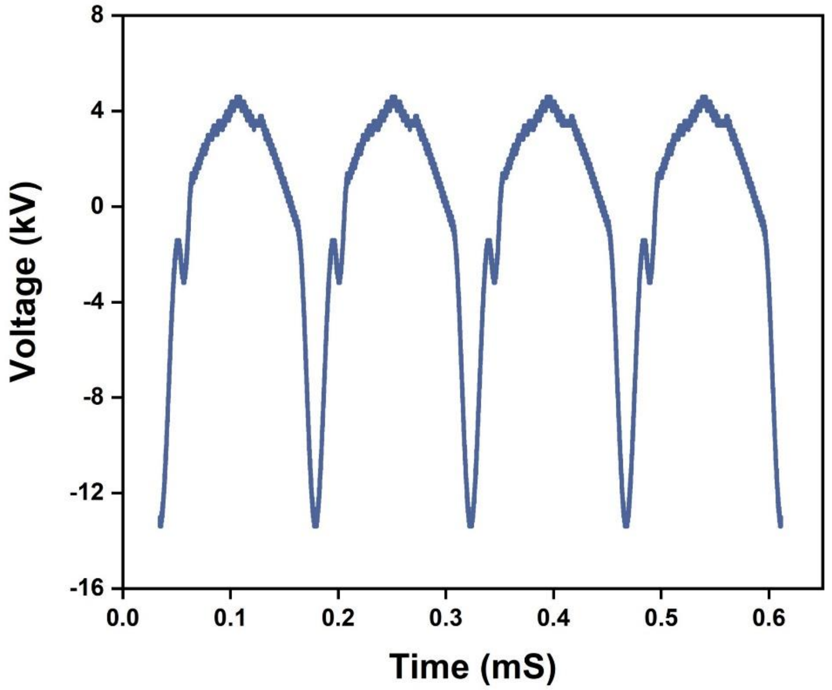

Figure S15. The voltage waveforms for the AC power supply.

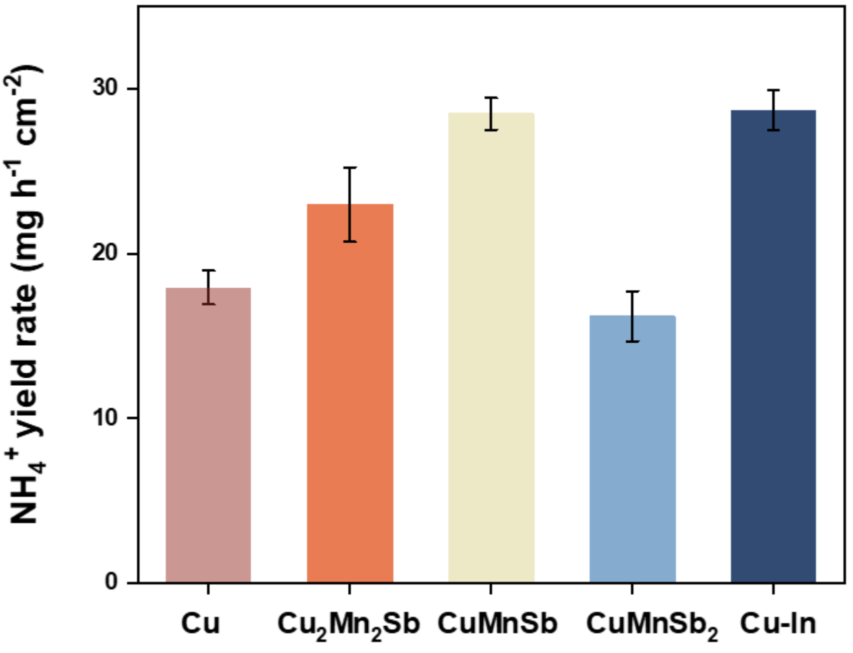

Figure S16. The NH_4^+ production rates of synthesized non-noble Cu based catalysts.

3.4 Calculation Structures

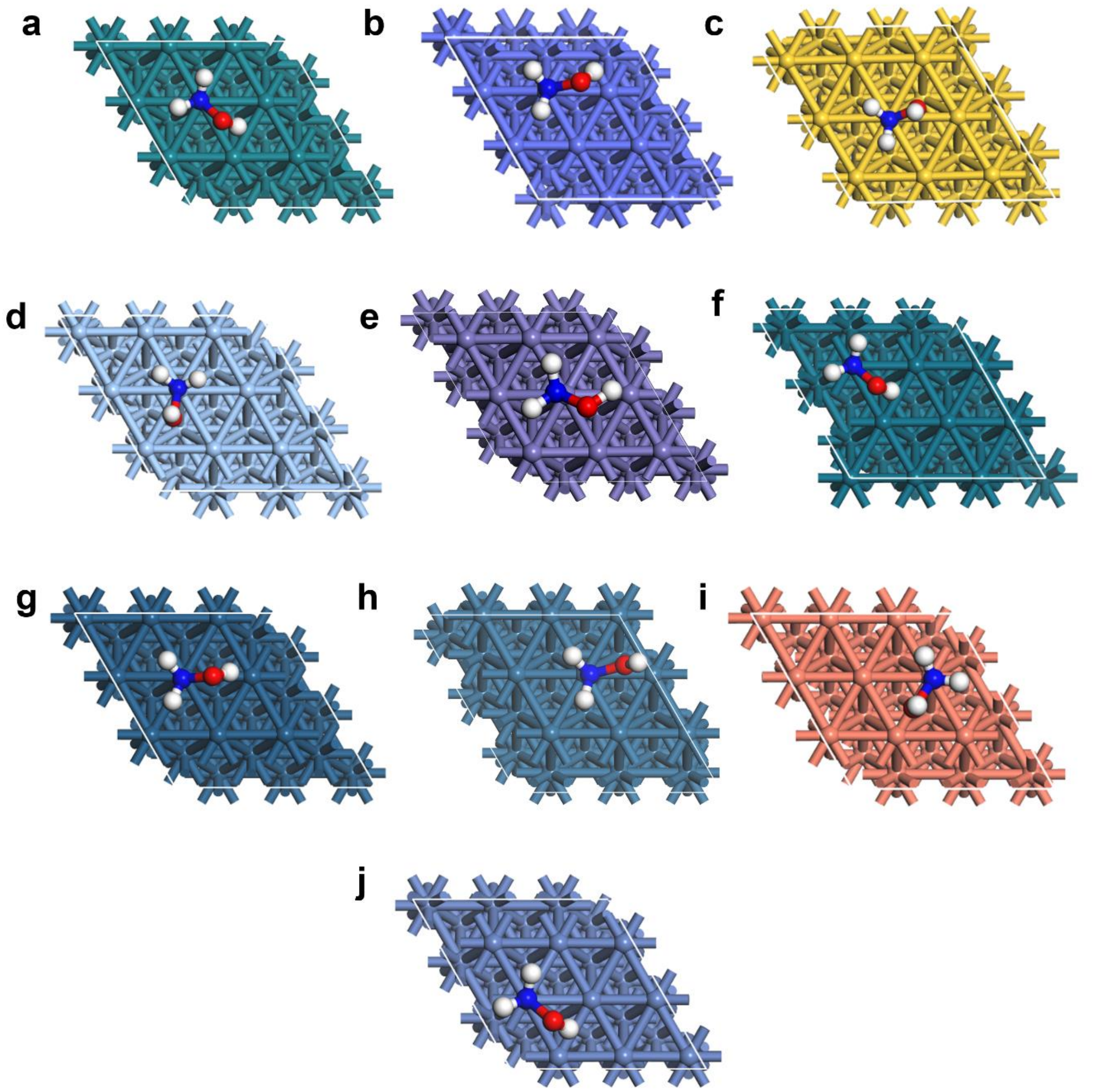

Figure S17. The optimized structures when NH_2OH absorbed on (a) Rh, (b) Co, (c) Au, (d) Ag, (e) Fe, (f) Pd, (g) Ir, (h) Os, (i) Cu, (j) Ni.

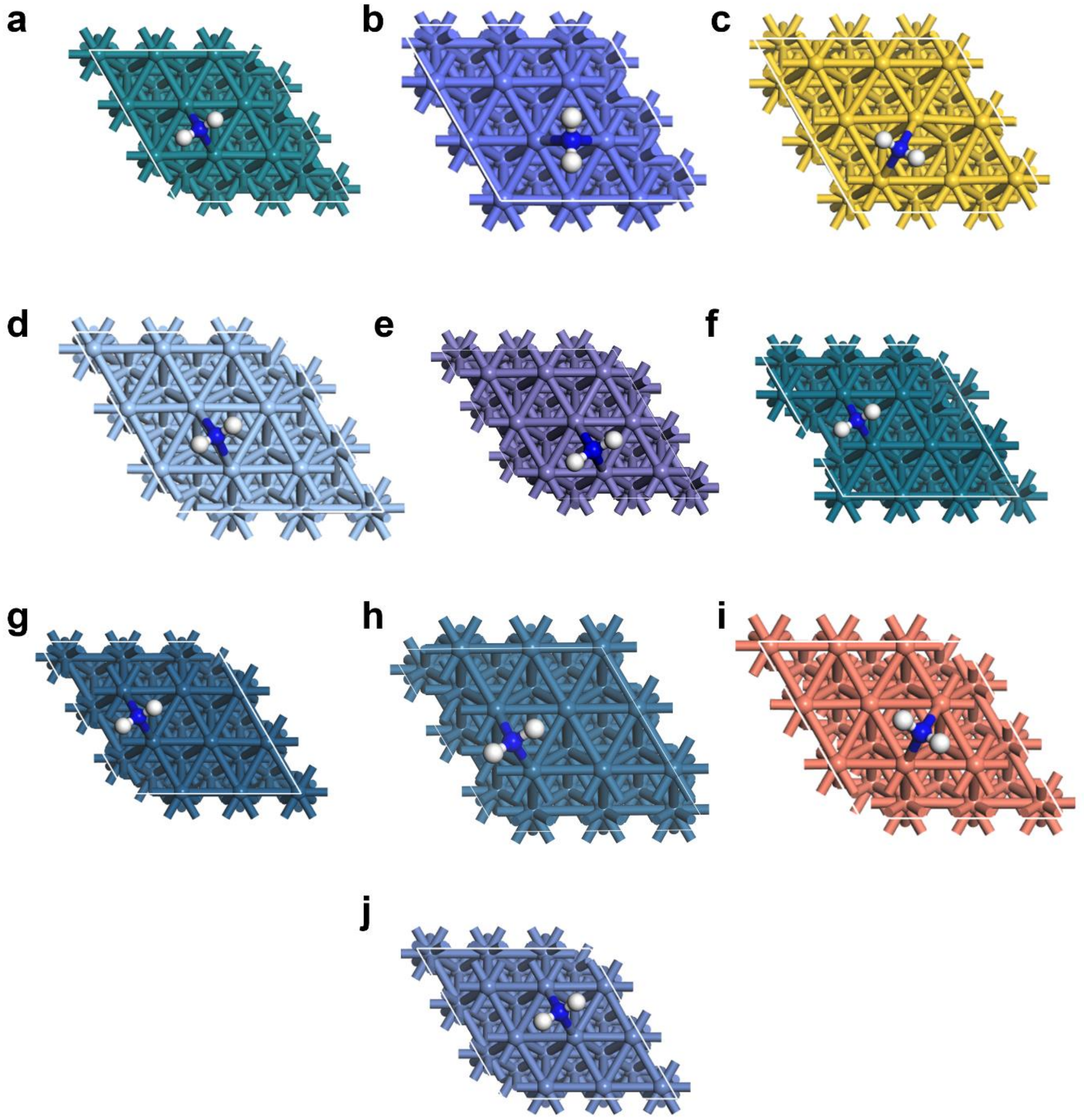

Figure S18. The optimized structures when NH_2 absorbed on (a) Rh, (b) Co, (c) Au, (d) Ag, (e) Fe, (f) Pd, (g) Ir, (h) Os, (i) Cu, (j) Ni.

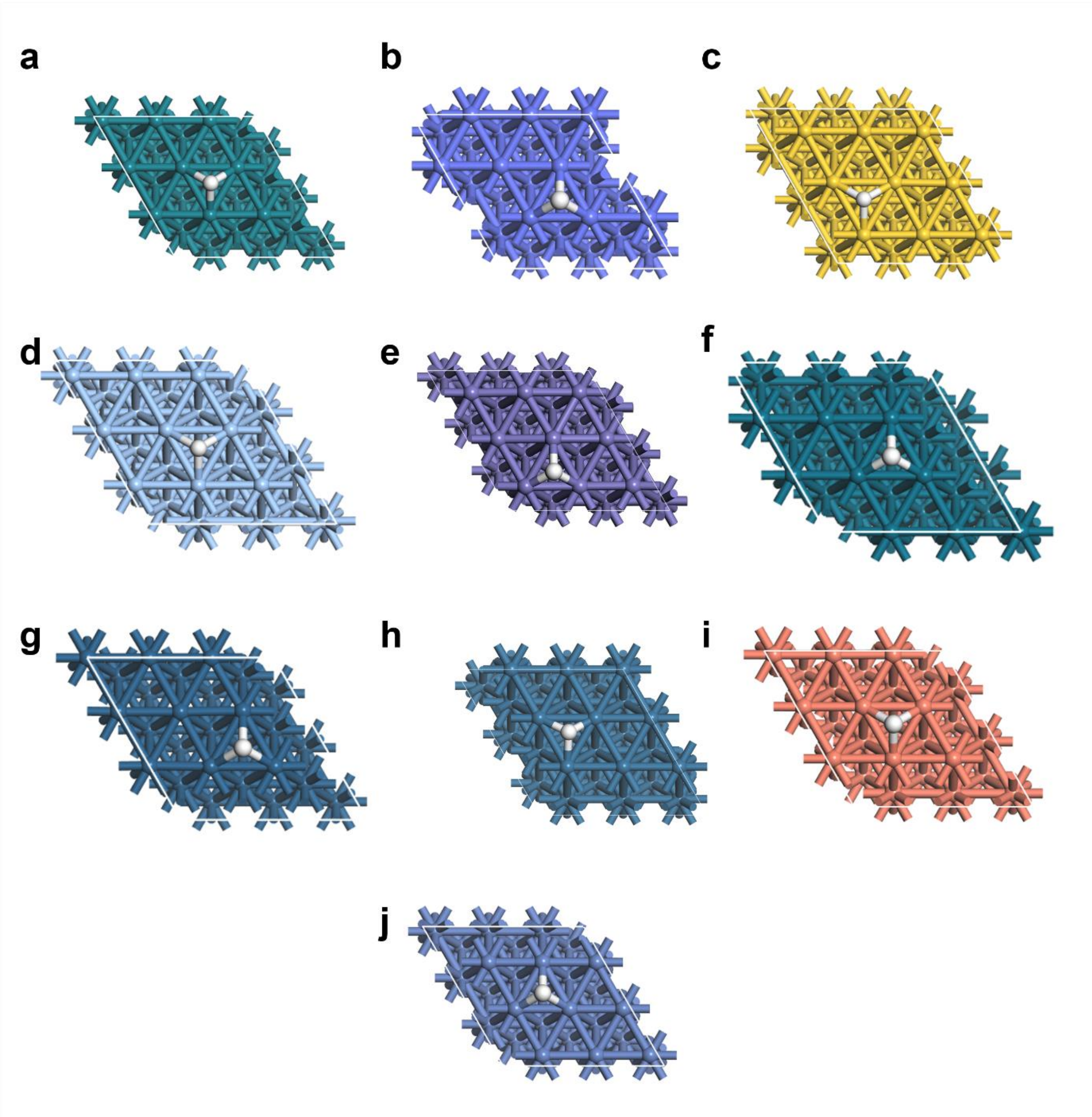

Figure S19. The optimized structures when H absorbed on (a) Rh, (b) Co, (c) Au, (d) Ag, (e) Fe, (f) Pd, (g) Ir, (h) Os, (i) Cu, (j) Ni.

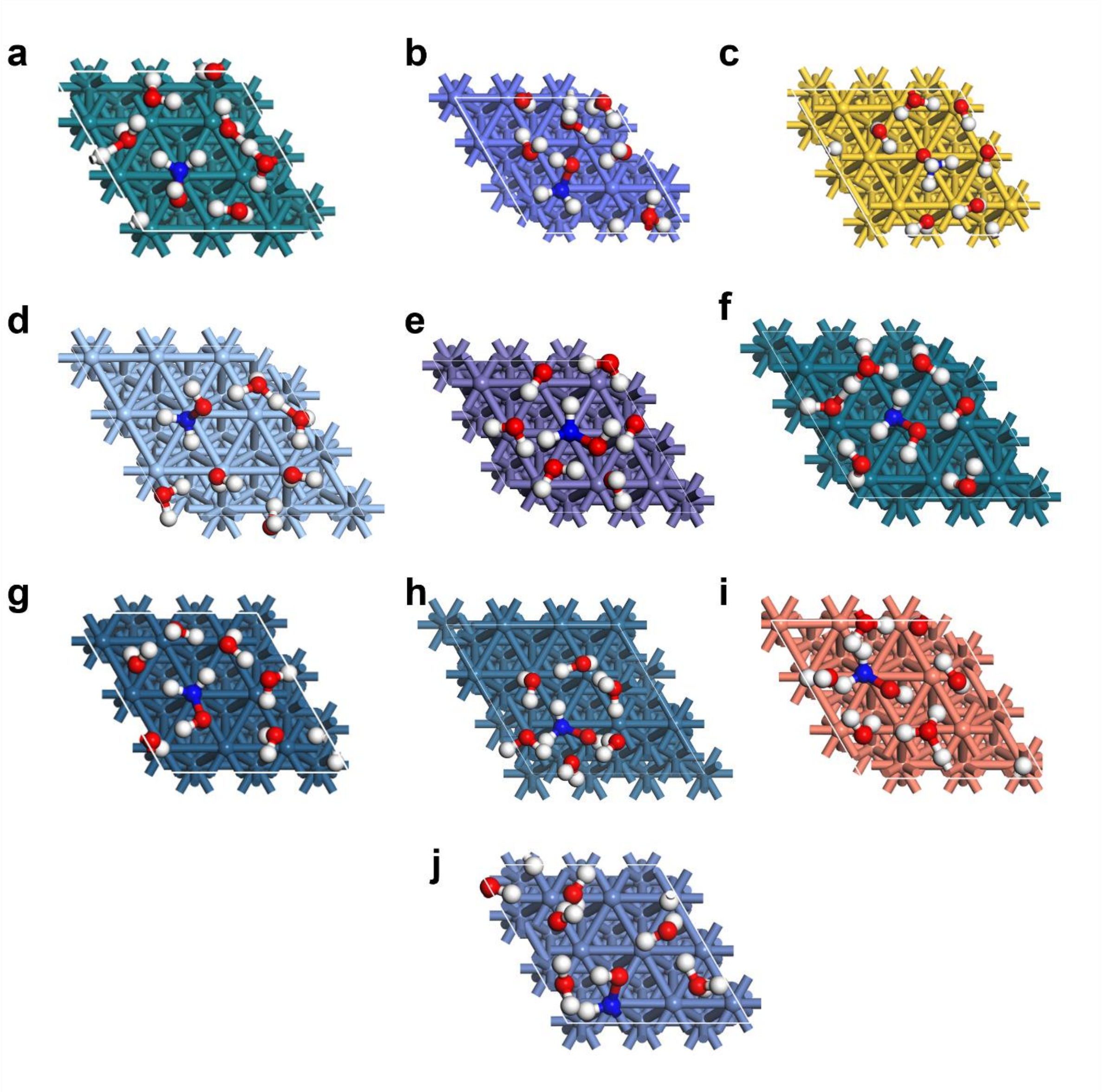

Figure S20. The optimized structures when NH_2OH adsorbed on (a) Rh, (b) Co, (c) Au, (d) Ag, (e) Fe, (f) Pd, (g) Ir, (h) Os, (i) Cu, (j) Ni in single-layer-water structures.

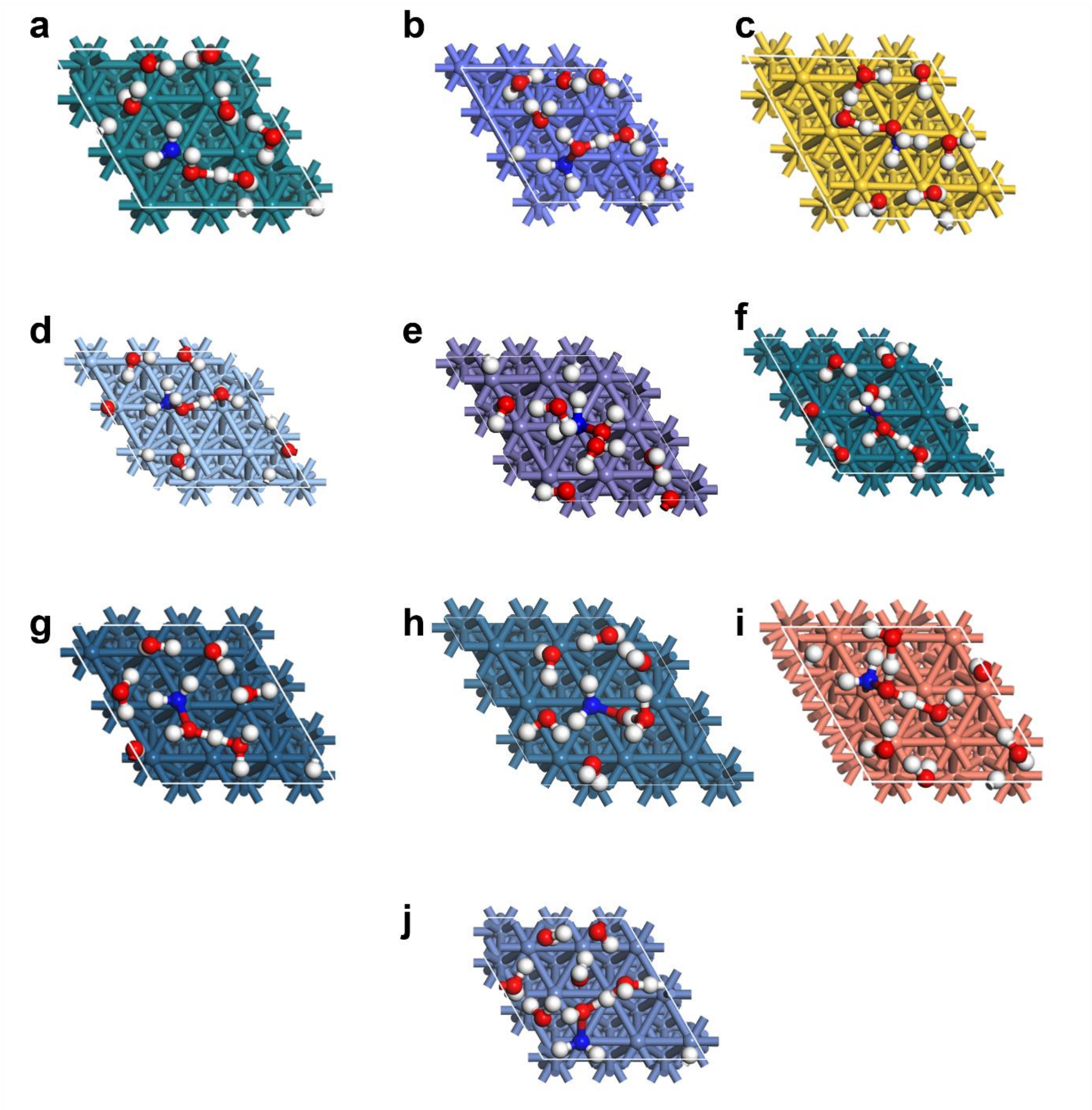

Figure S21. The transition state structures of hydrogenation of NH_2OH on (a) Rh, (b) Co, (c) Au, (d) Ag, (e) Fe, (f) Pd, (g) Ir, (h) Os, (i) Cu, (j) Ni in single-layer-water structures by ER mechanism.

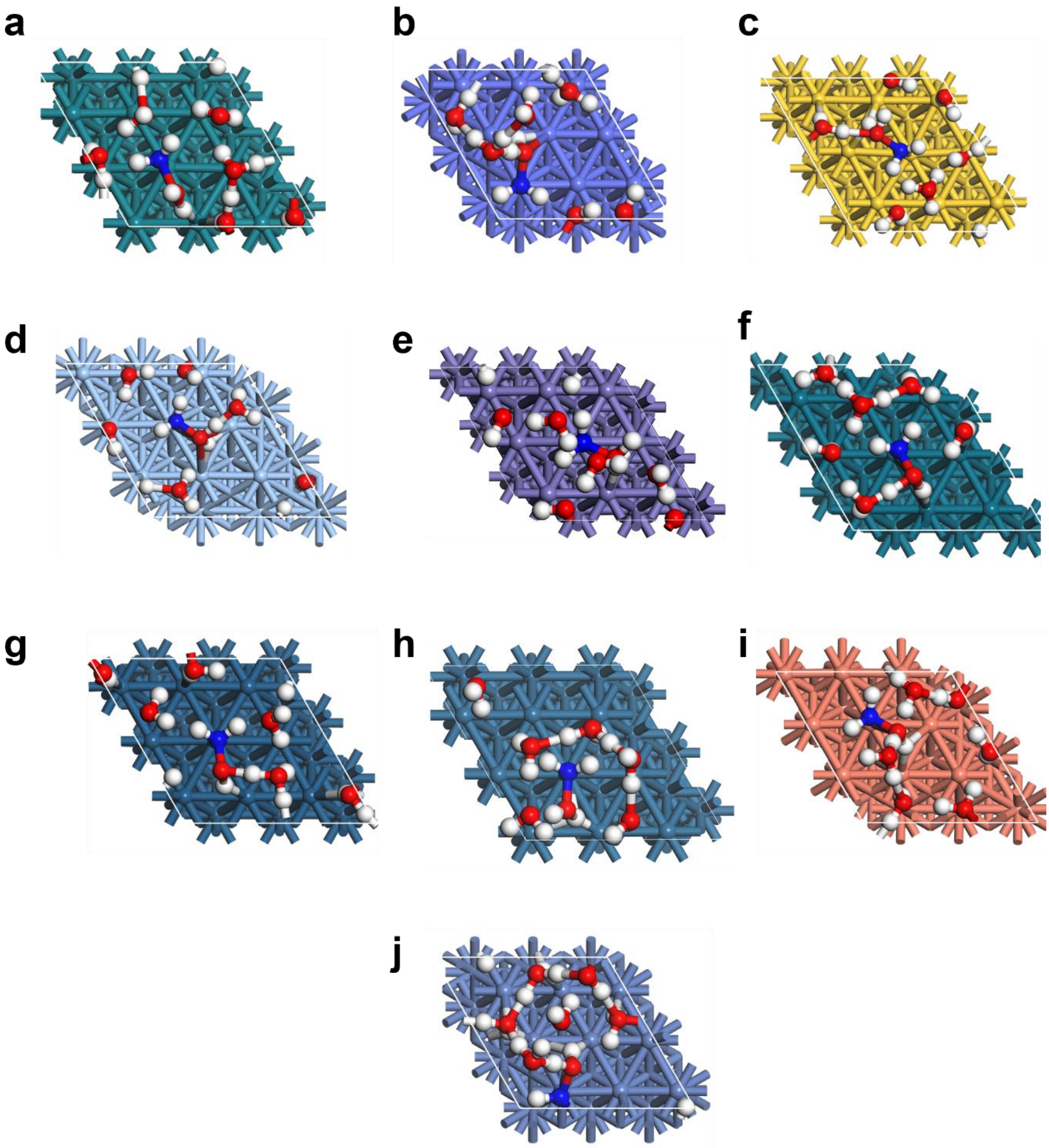

Figure S22. The transition state structures of hydrogenation of NH_2OH on (a) Rh, (b) Co, (c) Au, (d) Ag, (e) Fe, (f) Pd, (g) Ir, (h) Os, (i) Cu, (j) Ni in single-layer-water structures by LH mechanism.

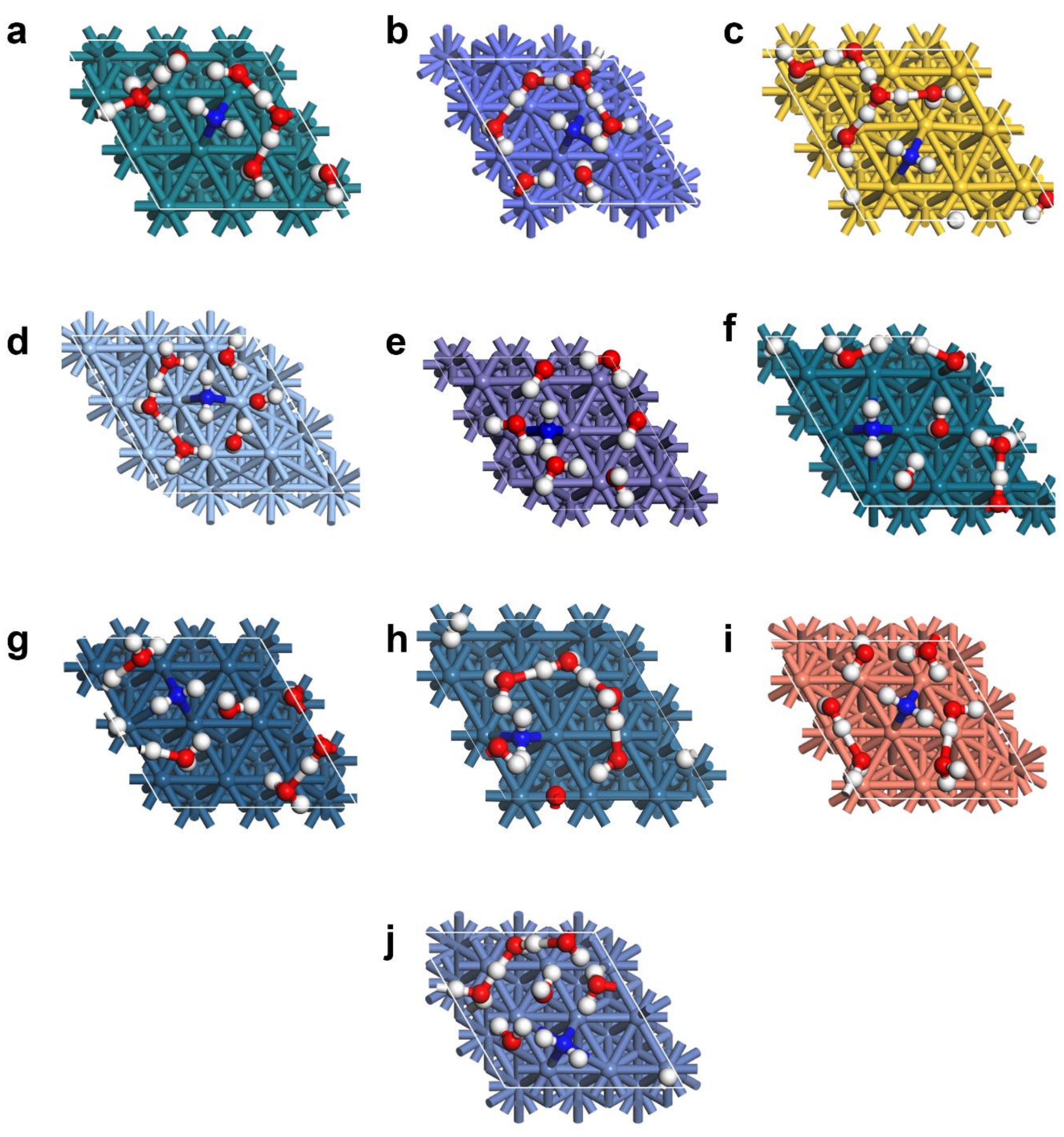

Figure S23. The optimized structures when NH_2 adsorbed on (a) Rh, (b) Co, (c) Au, (d) Ag, (e) Fe, (f) Pd, (g) Ir, (h) Os, (i) Cu, (j) Ni in single-layer-water structures.

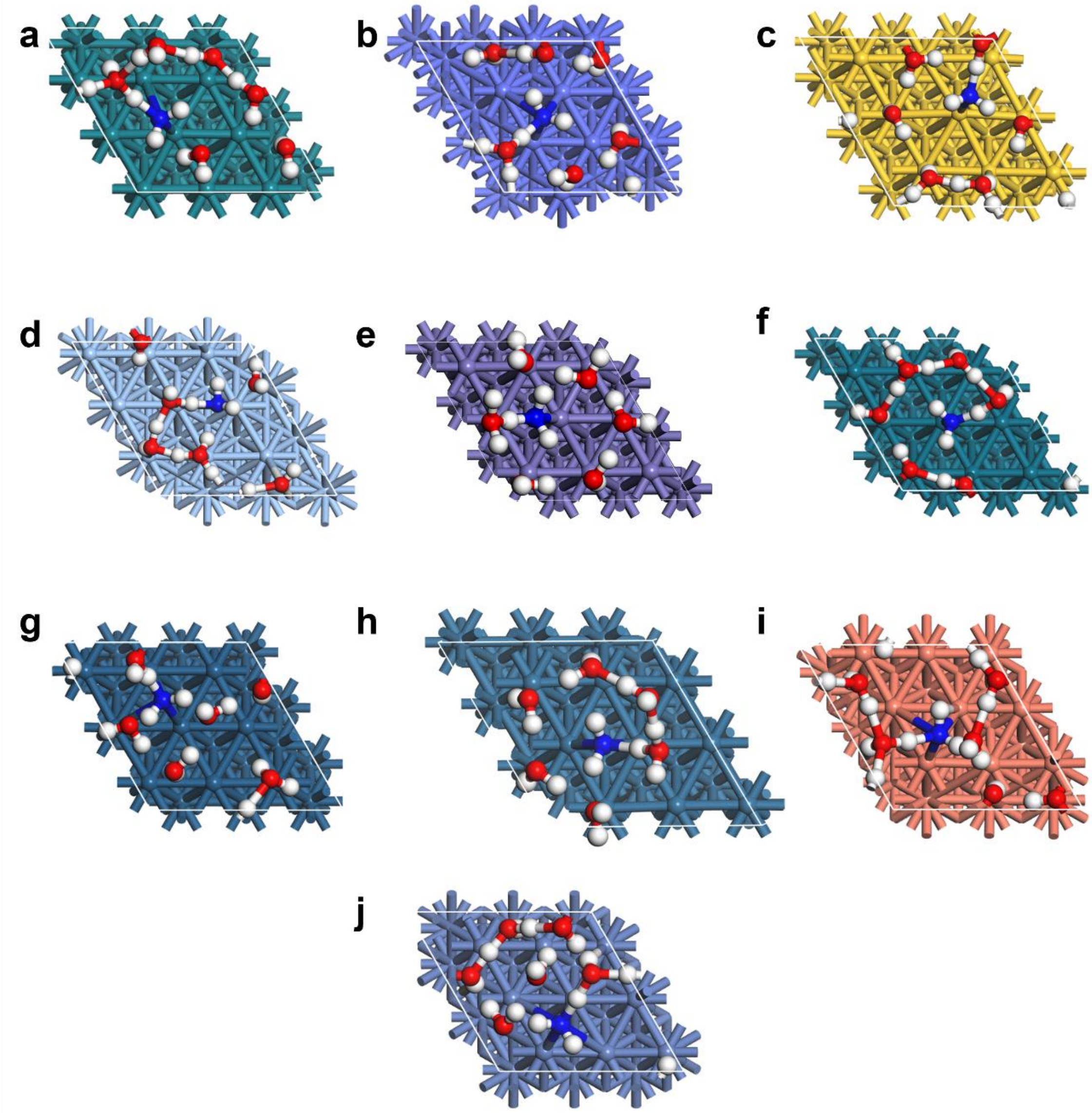

Figure S24. The transition state structures of hydrogenation of NH_2 on (a) Rh, (b) Co, (c) Au, (d) Ag, (e) Fe, (f) Pd, (g) Ir, (h) Os, (i) Cu, (j) Ni in single-layer-water structures by ER mechanism.

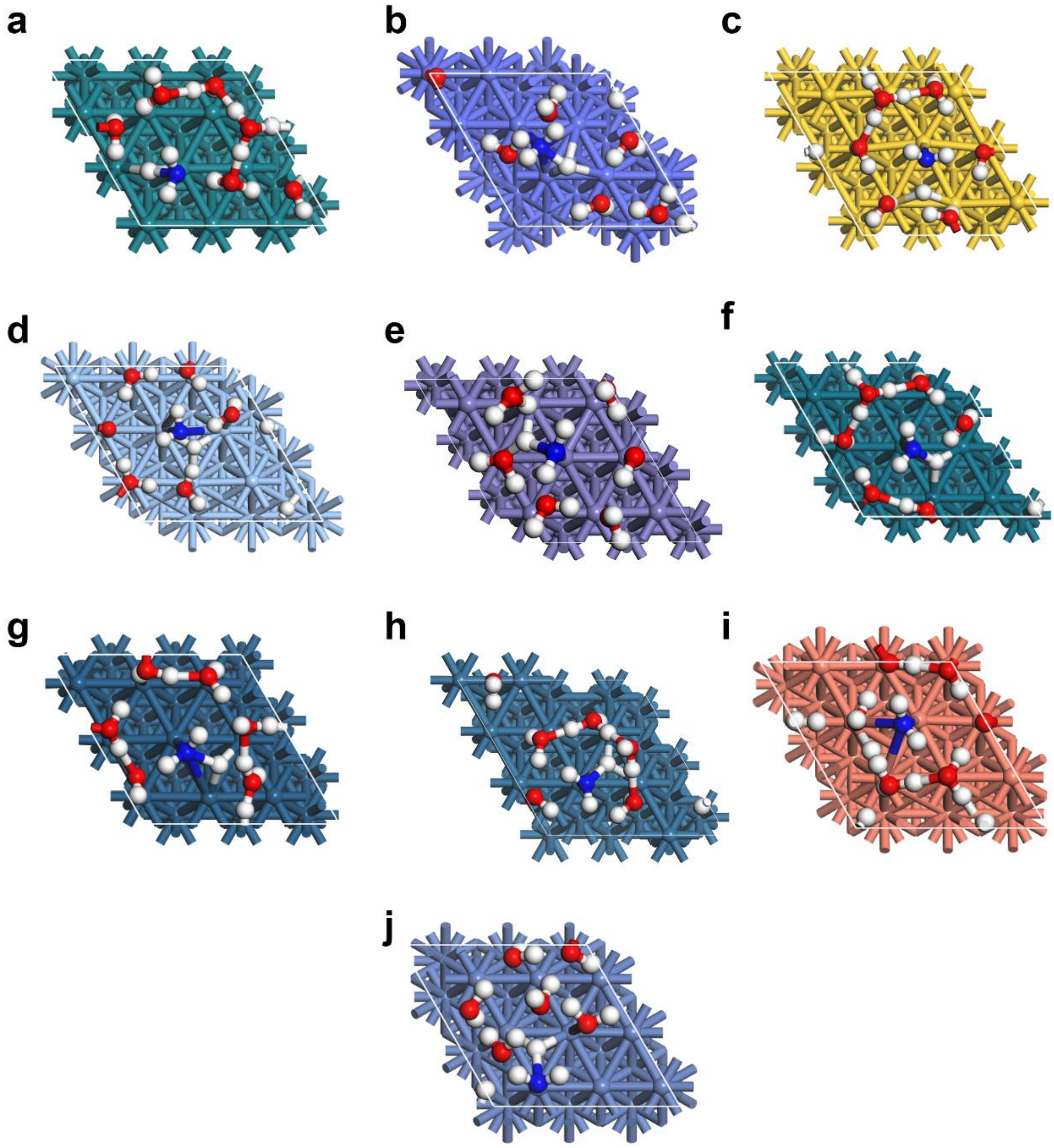

Figure S25. The transition state structures of hydrogenation of NH_2 on (a) Rh, (b) Co, (c) Au, (d) Ag, (e) Fe, (f) Pd, (g) Ir, (h) Os, (i) Cu, (j) Ni in single-layer-water structures by LH mechanism.

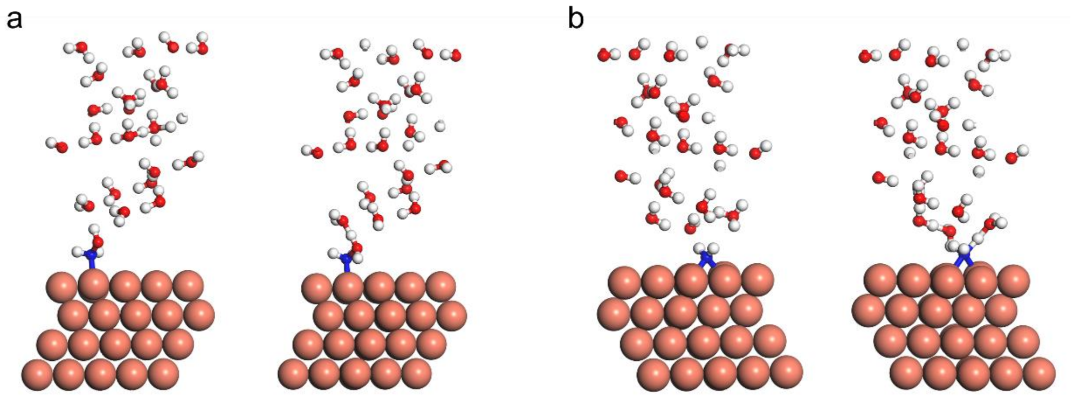

Figure S26. The initial and transition state of hydrogenation of (a) *NH_2OH, and (b) *NH_2 in the full solvent model.

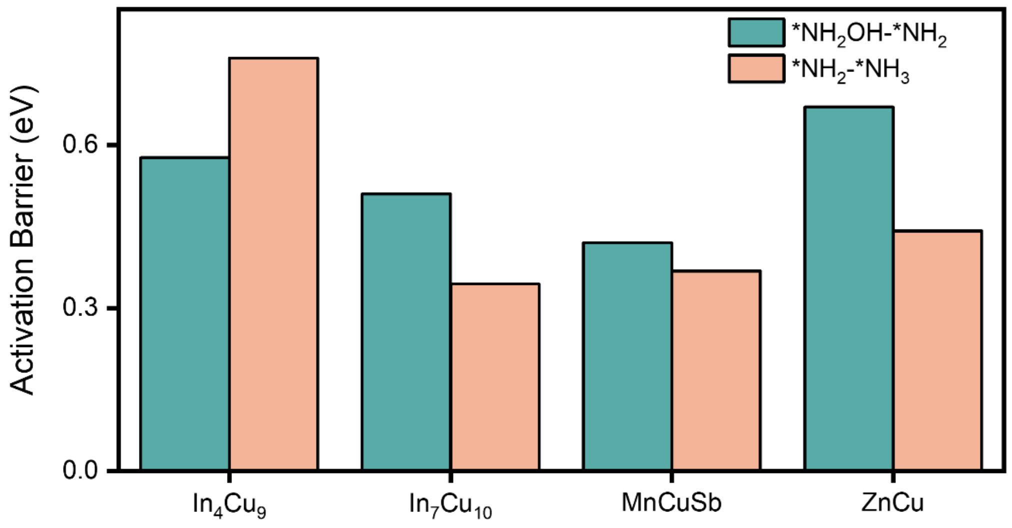

Figure S27. The activation barrier of the two hydrogenation steps on different candidates.

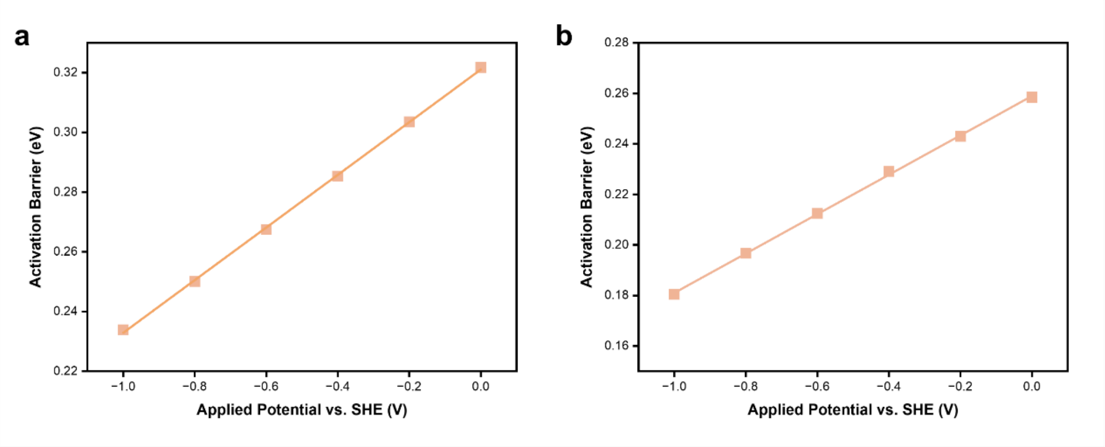

Figure S28. The activation barrier of $*NH_2OH$ to $*NH_2$, and $*NH_2$ to NH_3 on Cu slab under different potential vs. SHE.

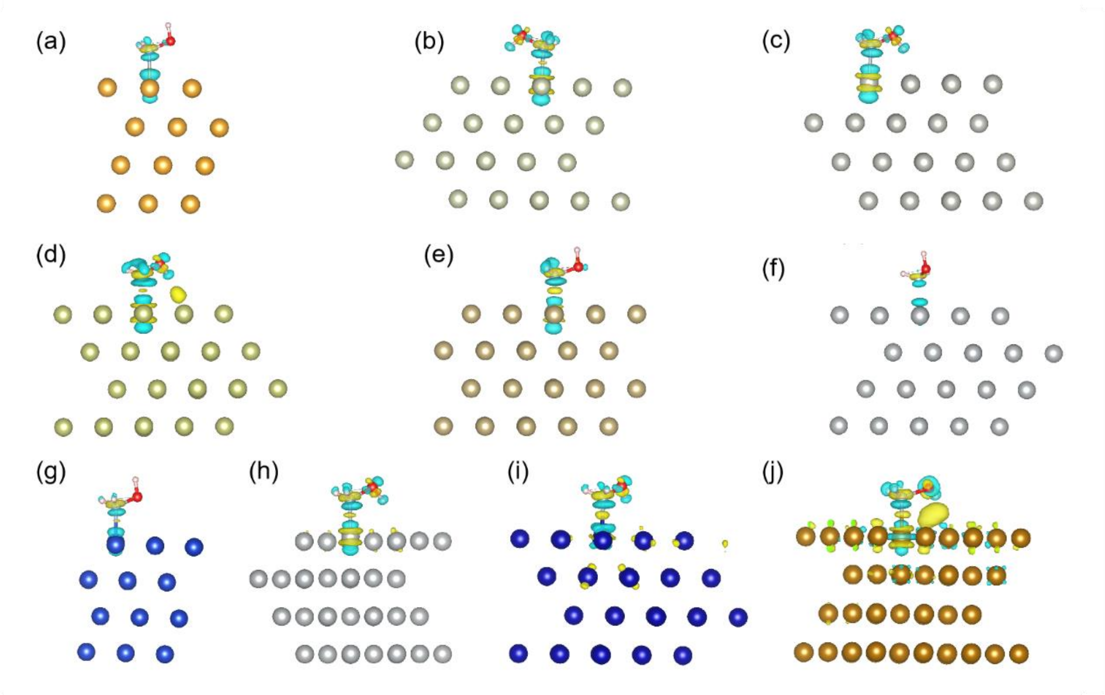

Figure S29. The charge density difference plot when NH_2OH is adsorbed on (a) Au, (b) Rh, (c) Pd, (d) Ir, (e) Os, (f) Ag, (g) Cu, (h) Ni, (i) Co, and (j) Fe, individually.

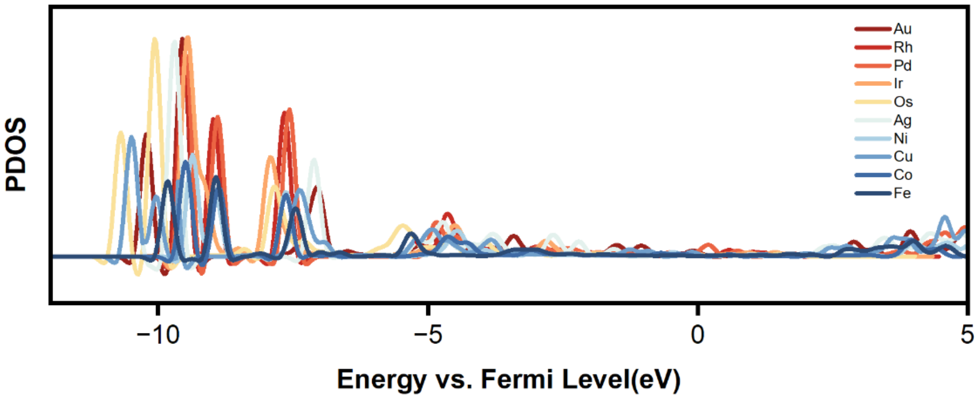

Figure S30. The projected density of states of p-orbital of N atom of NH_2OH on different slab.

3.5 Microkinetic flow chart under different NH_2 and H adsorption energy

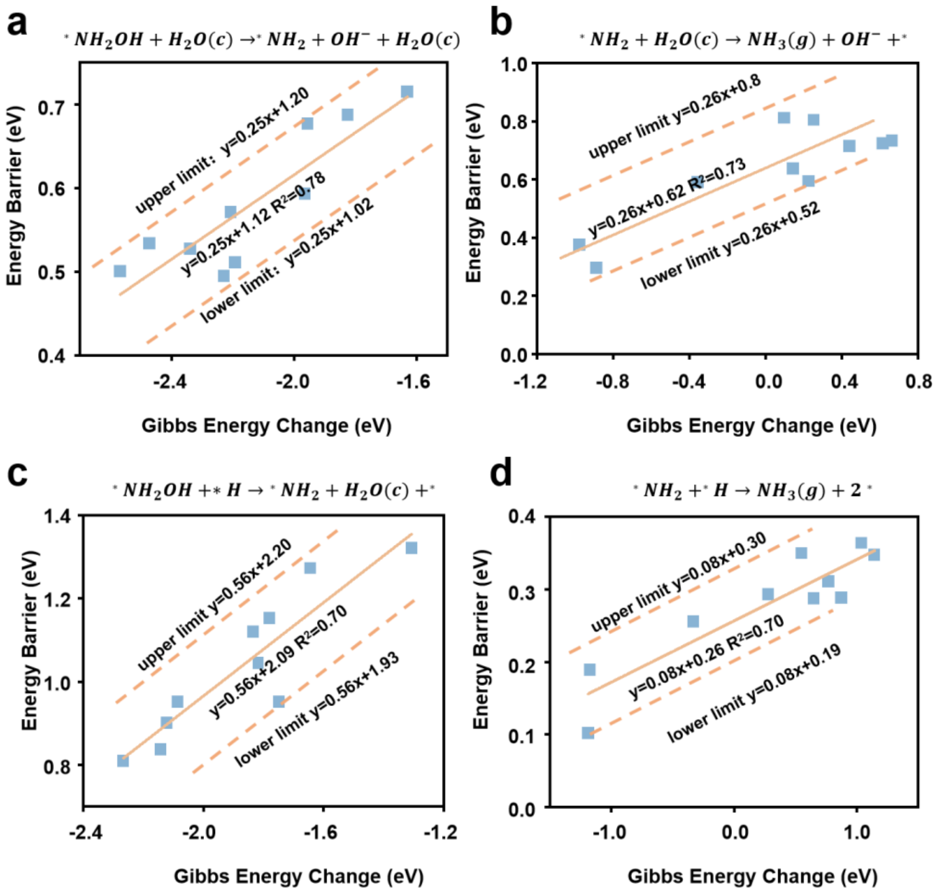

Figure S31. The upper limit and the lower limit of the BEP relationships of four elemental reactions.

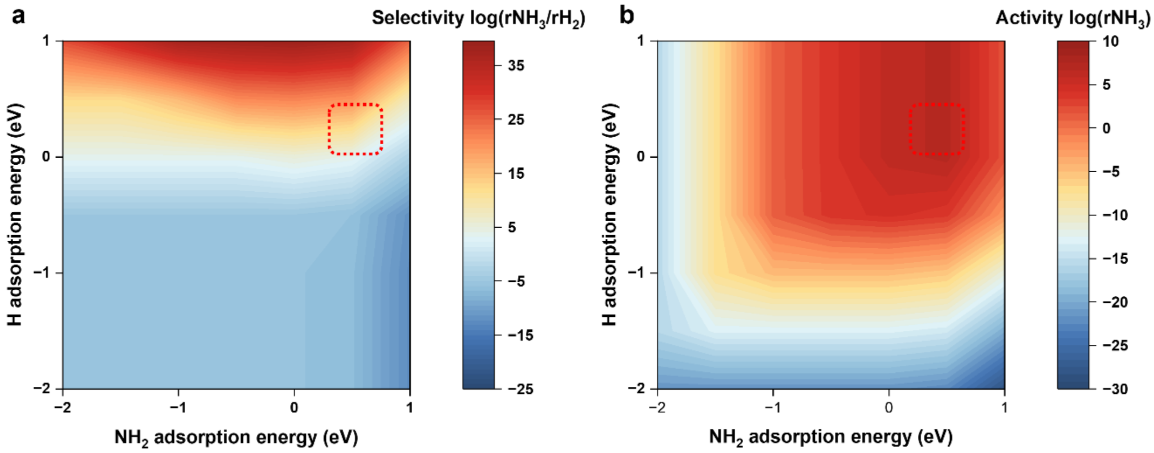

Figure S32. (a) The selectivity of NH_3 vs H_2 and (b) activity towards NH_3 for different NH_2 and H adsorption energy by the lower limit BEP relationship.

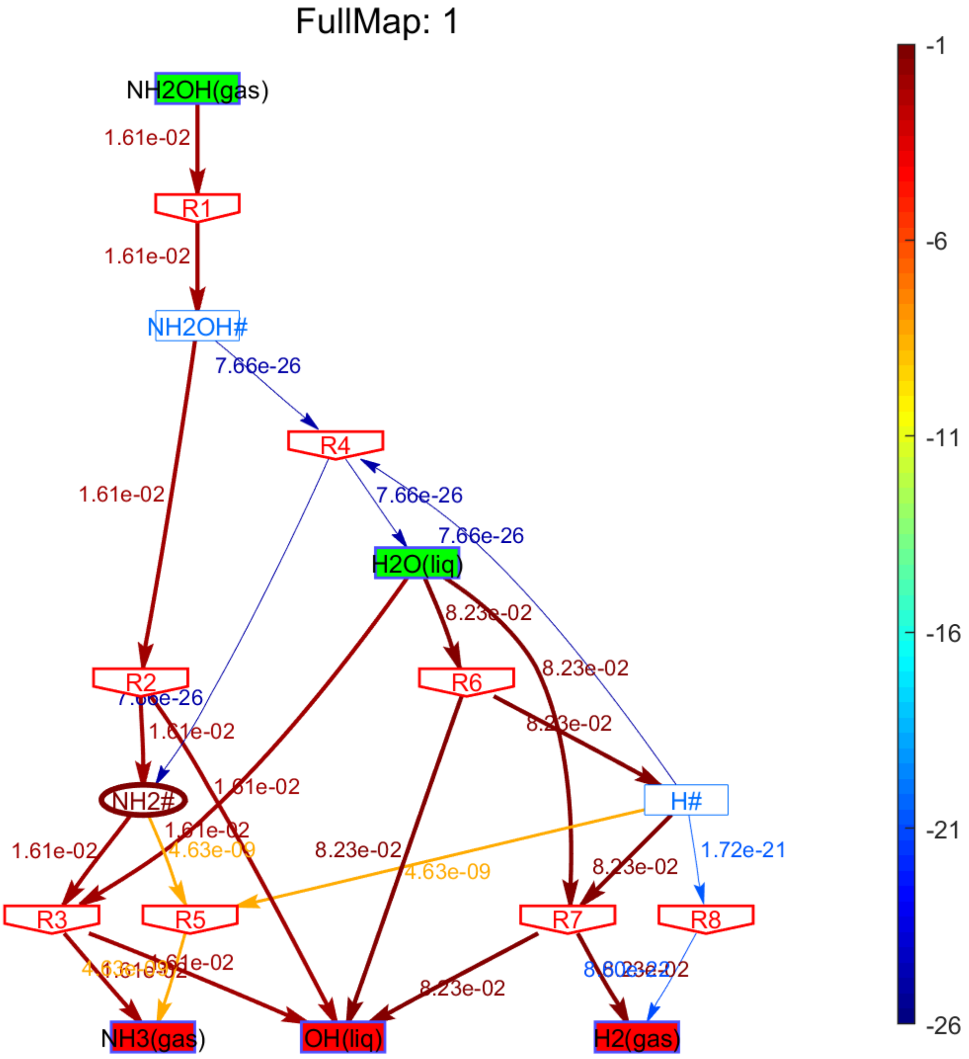

Figure S33. The full flow chart of NH_2OH reduction at NH_2 adsorption of -0.25 eV and H adsorption energy of -0.25 eV of upper limit.

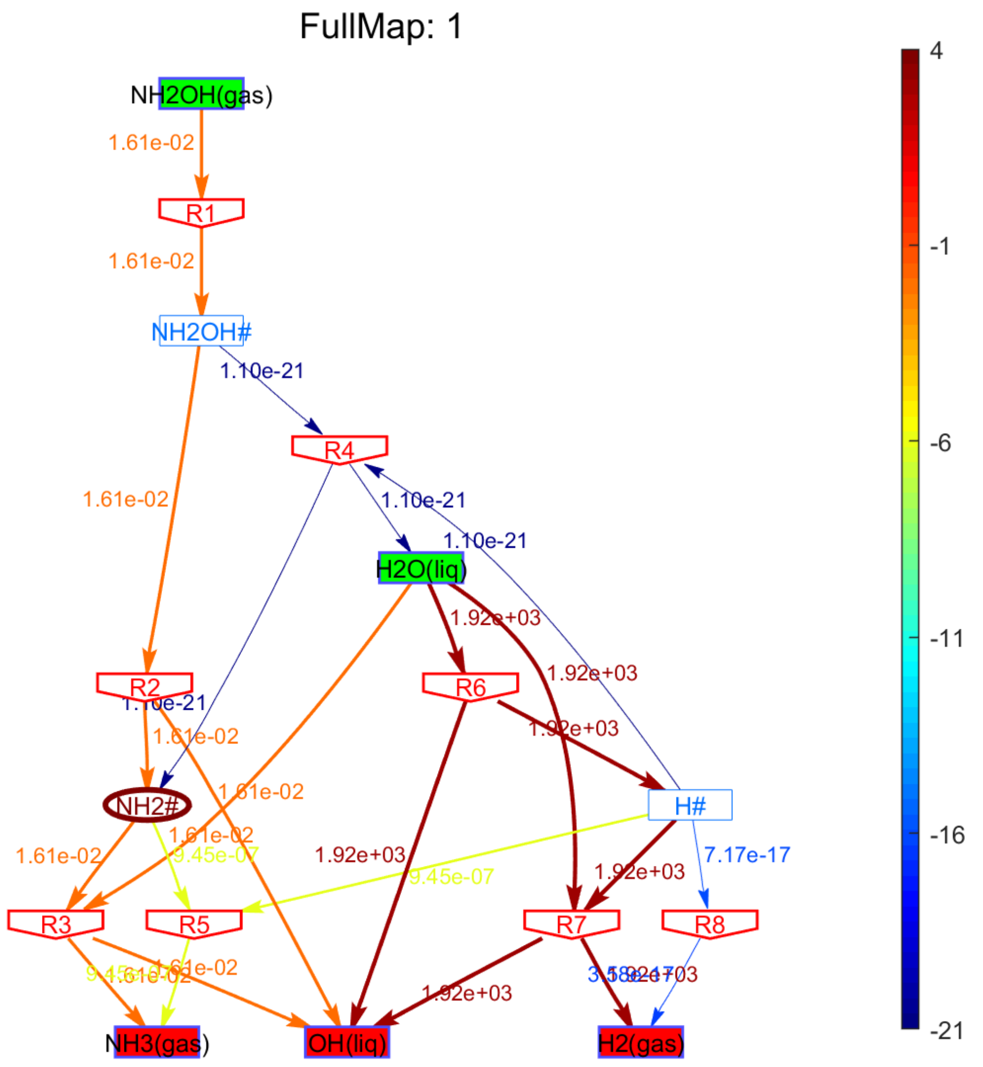

Figure S34. The full flow chart of NH_2OH reduction at NH_2 adsorption of -0.25 eV and H adsorption energy of -0.5 eV.

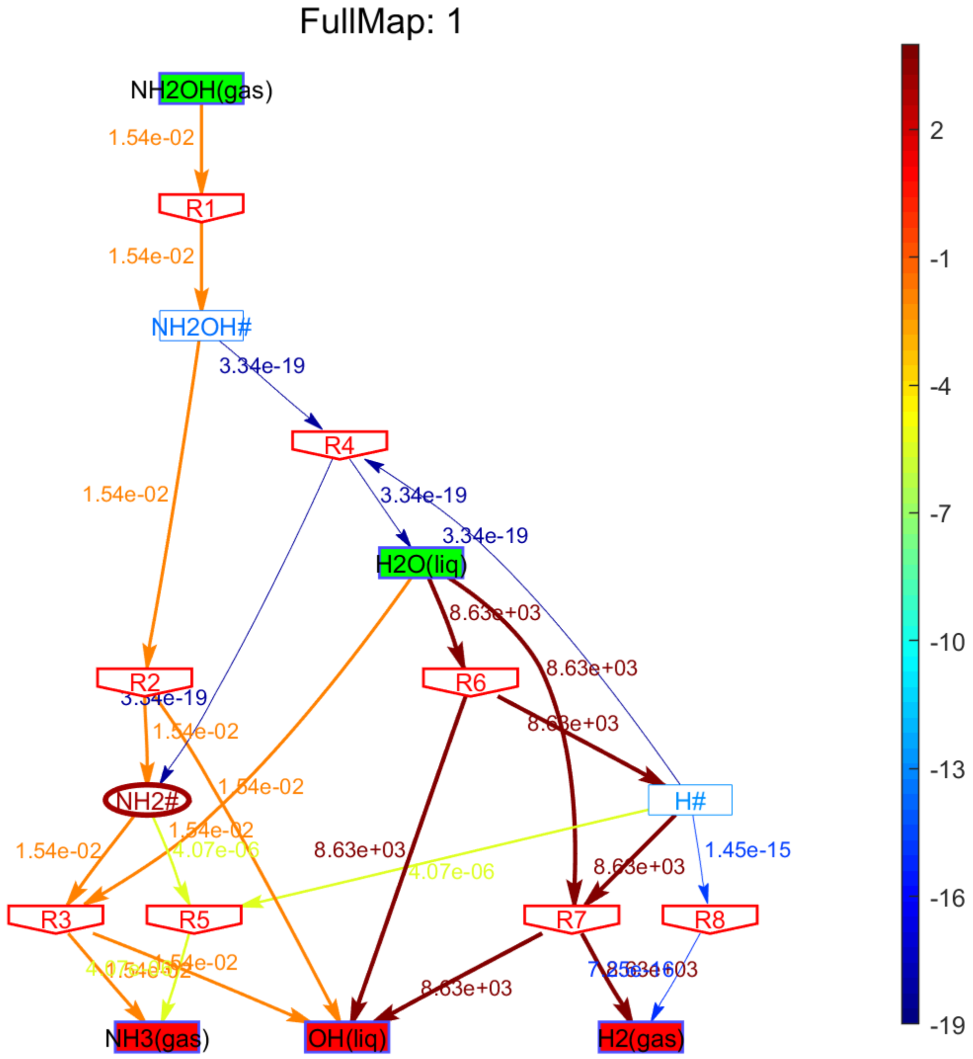

Figure S35. The full flow chart of NH_2OH reduction at NH_2 adsorption of -0.25 eV and H adsorption energy of -0.75 eV.

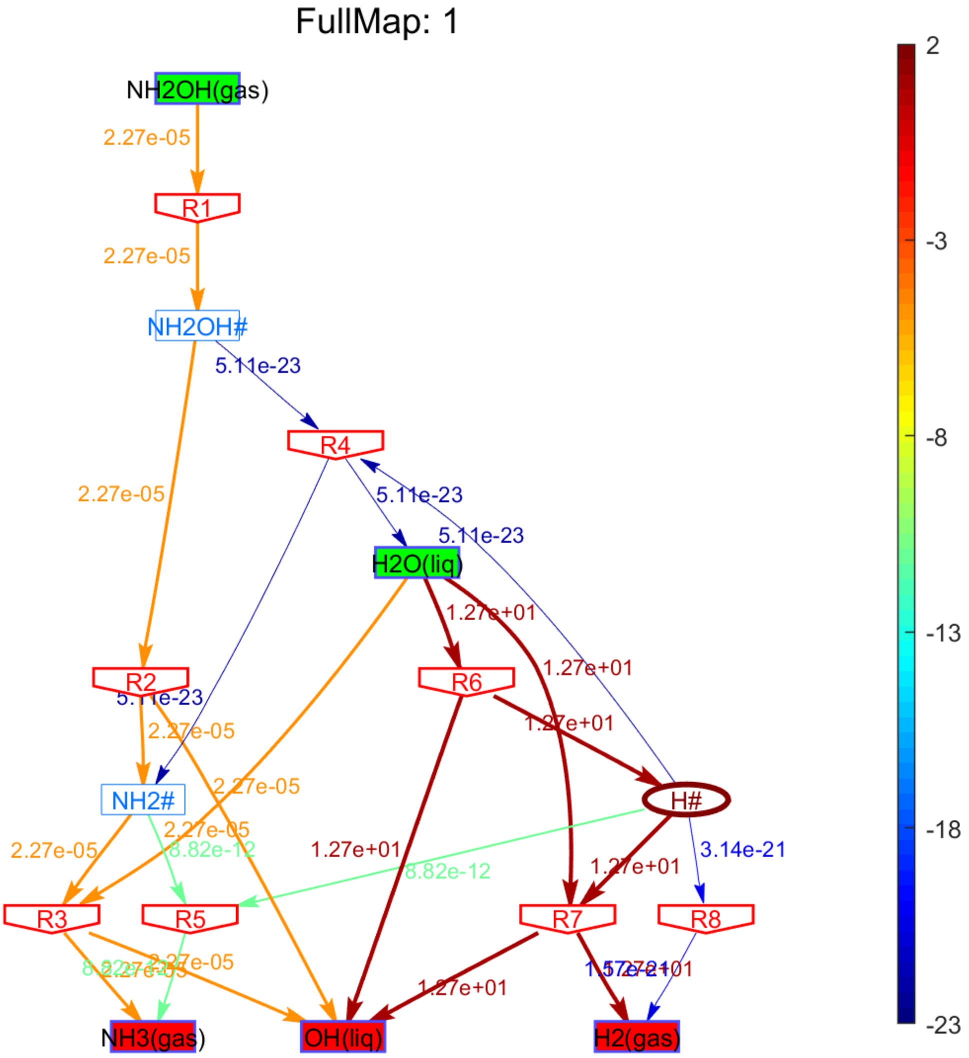

Figure S36. The full flow chart of NH_2OH reduction at NH_2 adsorption of -0.25 eV and H adsorption energy of -1.0 eV.

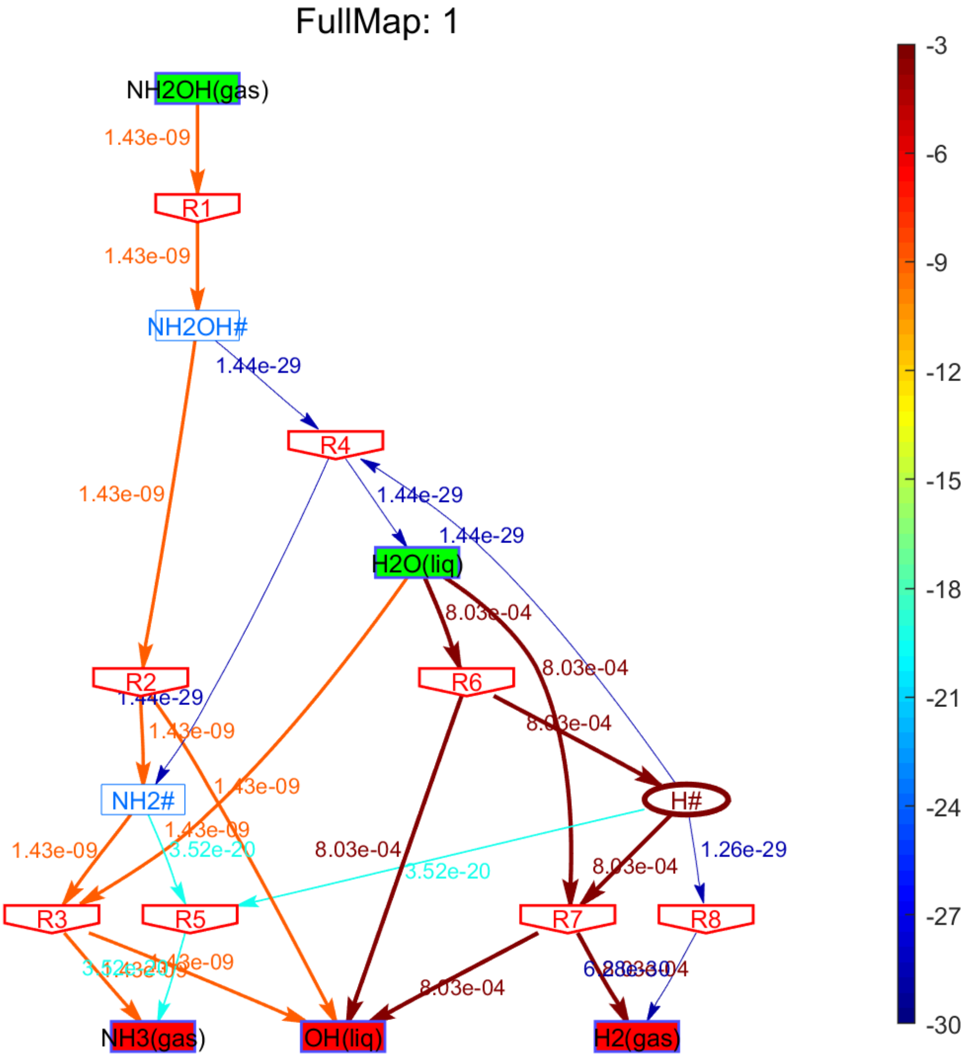

Figure S37. The full flow chart of NH_2OH reduction at NH_2 adsorption of -0.25 eV and H adsorption energy of -1.25 eV.

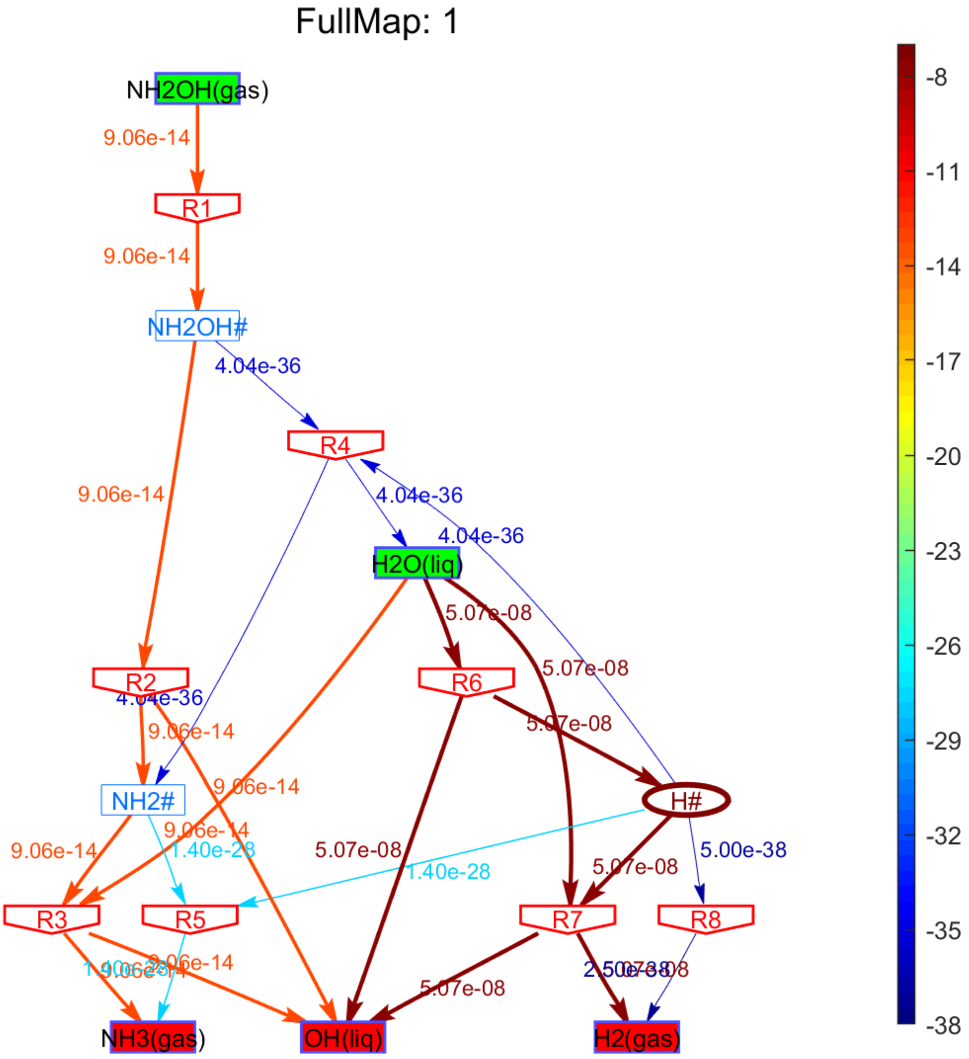

Figure S38. The full flow chart of NH_2OH reduction at NH_2 adsorption of -0.25 eV and H adsorption energy of -1.5 eV.

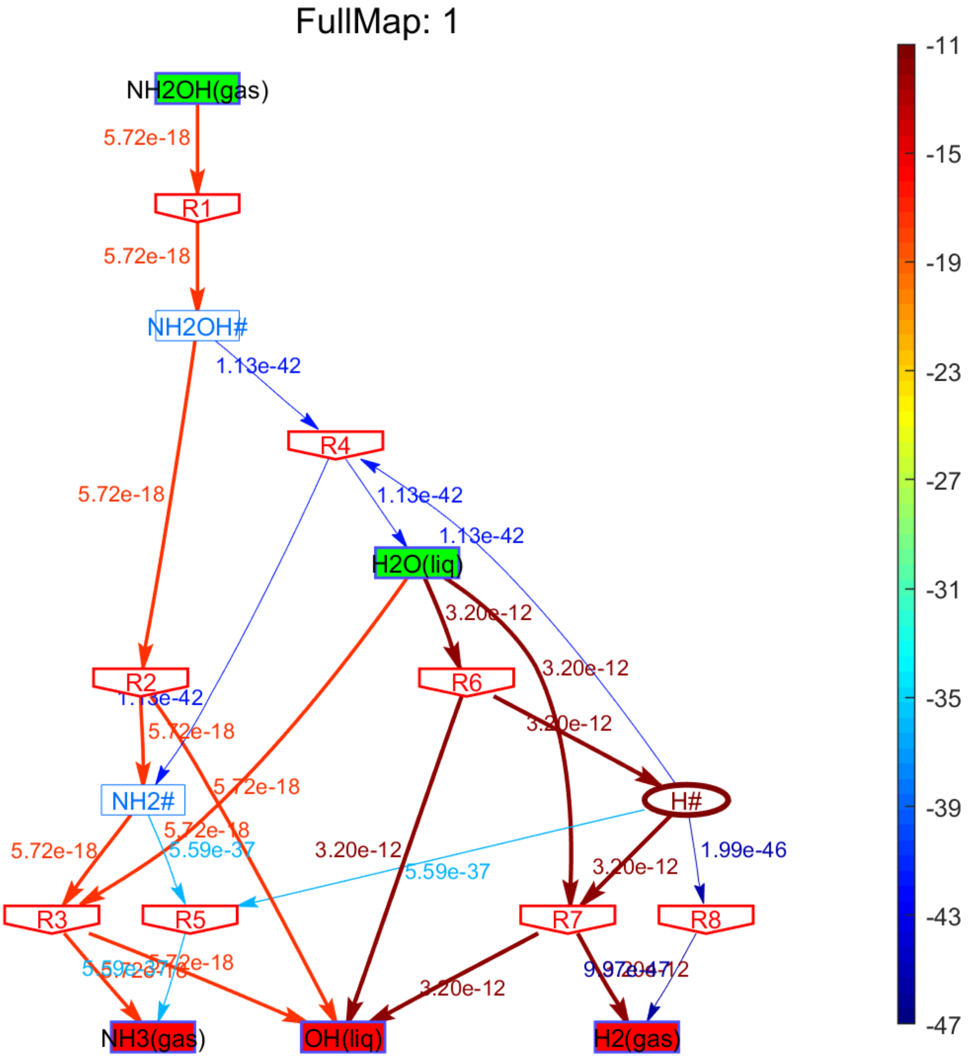

Figure S39. The full flow chart of NH_2OH reduction at NH_2 adsorption of -0.25 eV and H adsorption energy of -1.75 eV.

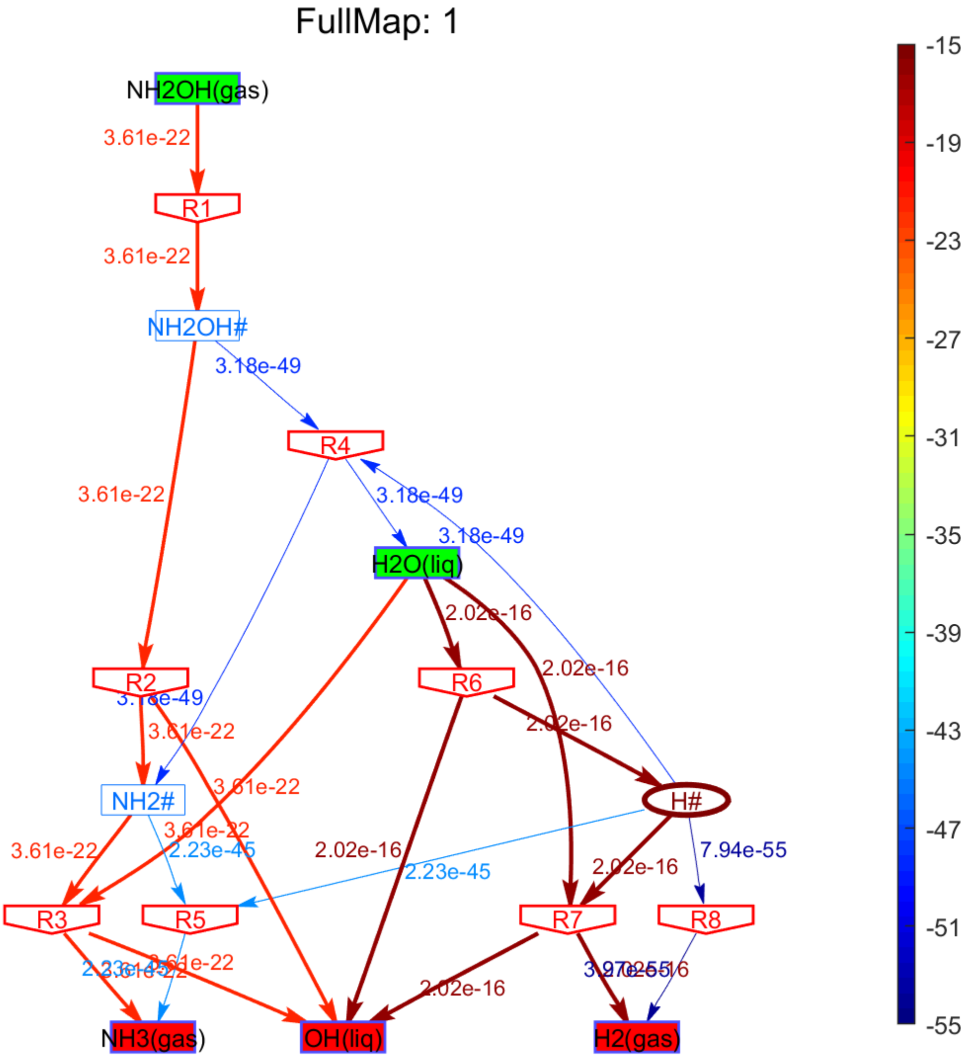

Figure S40. The full flow chart of NH_2OH reduction at NH_2 adsorption of -0.25 eV and H adsorption energy of -2.0 eV.

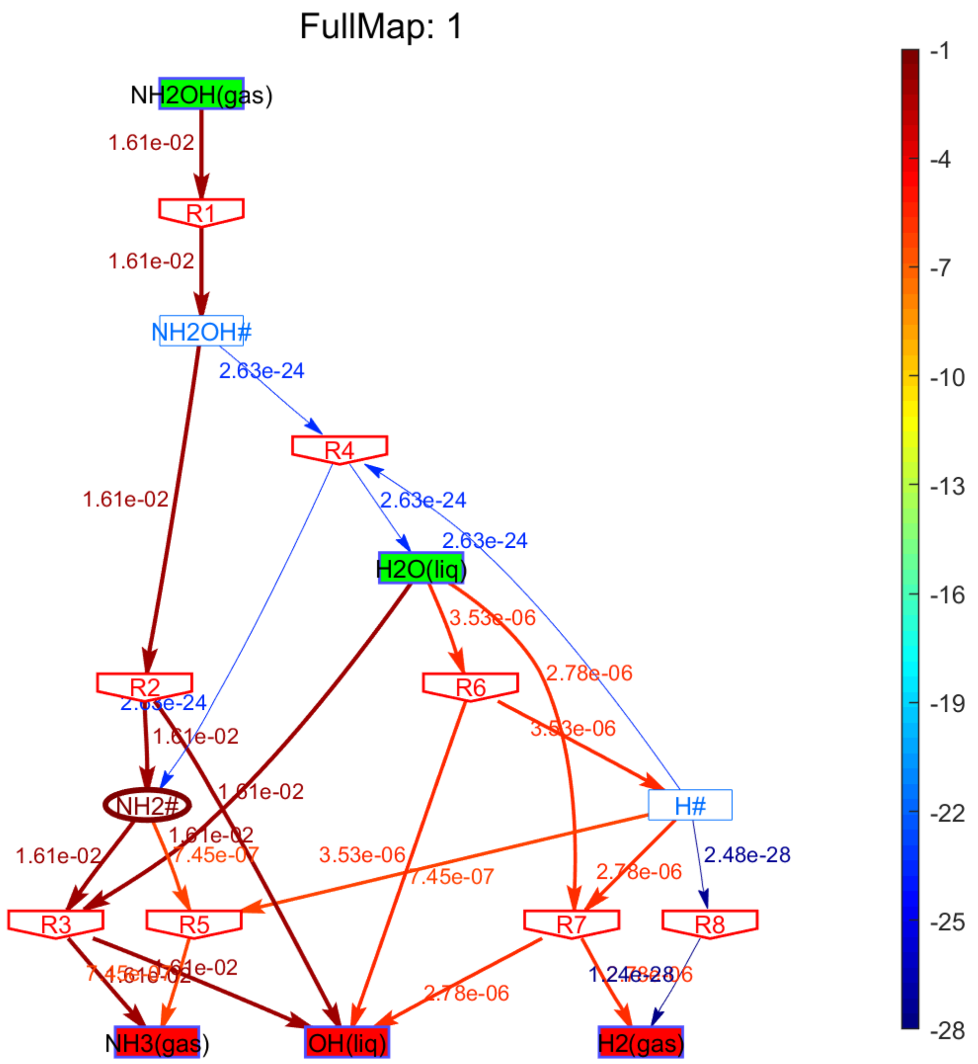

Figure S41. The full flow chart of NH_2OH reduction at NH_2 adsorption of -0.25 eV and H adsorption energy of 0.0 eV.

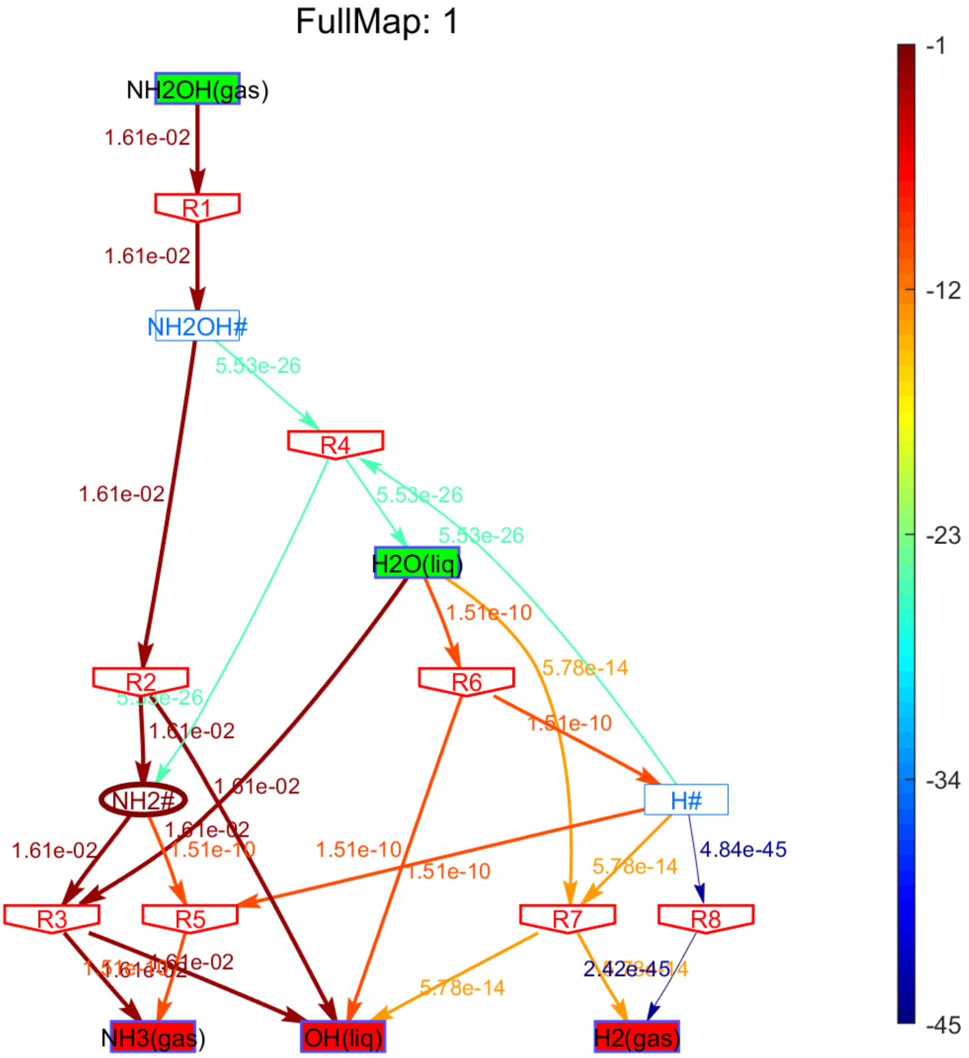

Figure S42. The full flow chart of NH_2OH reduction at NH_2 adsorption of -0.25 eV and H adsorption energy of 0.25 eV.

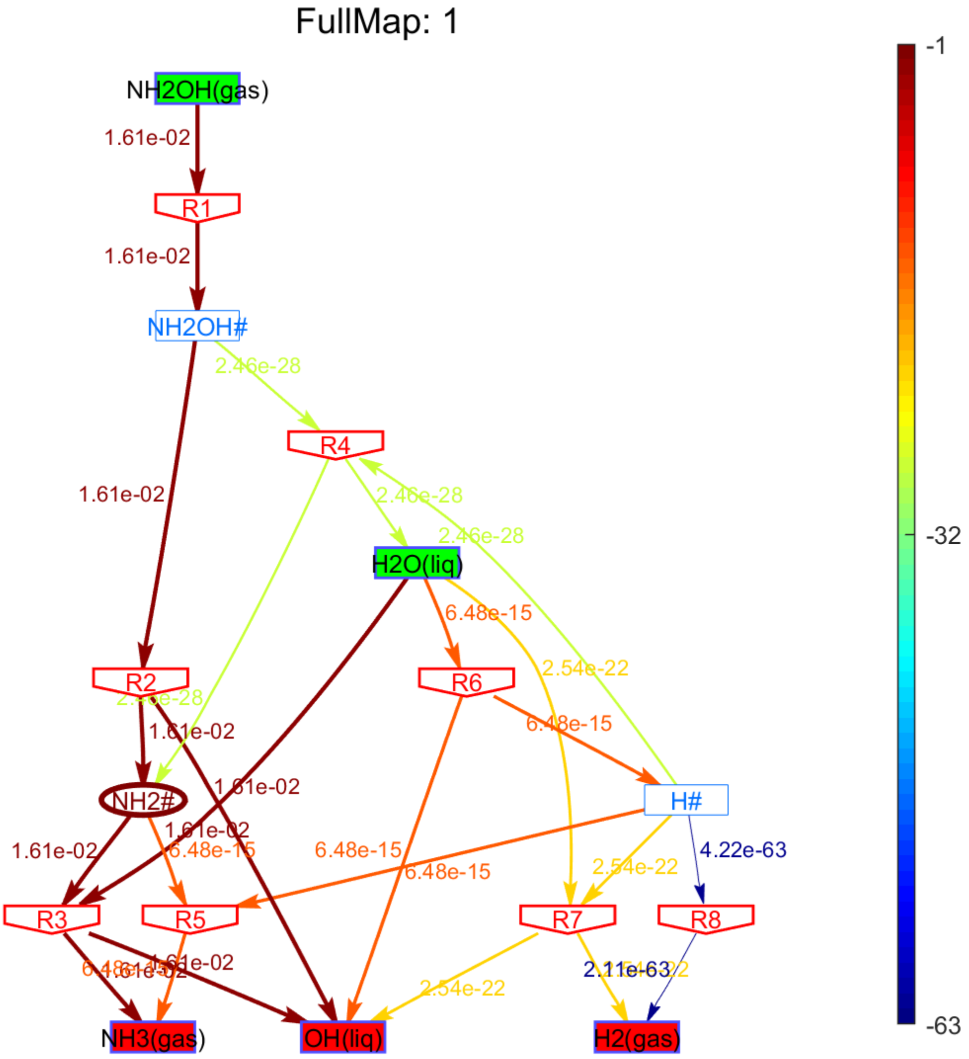

Figure S43. The full flow chart of NH_2OH reduction at NH_2 adsorption of -0.25 eV and H adsorption energy of 0.5 eV.

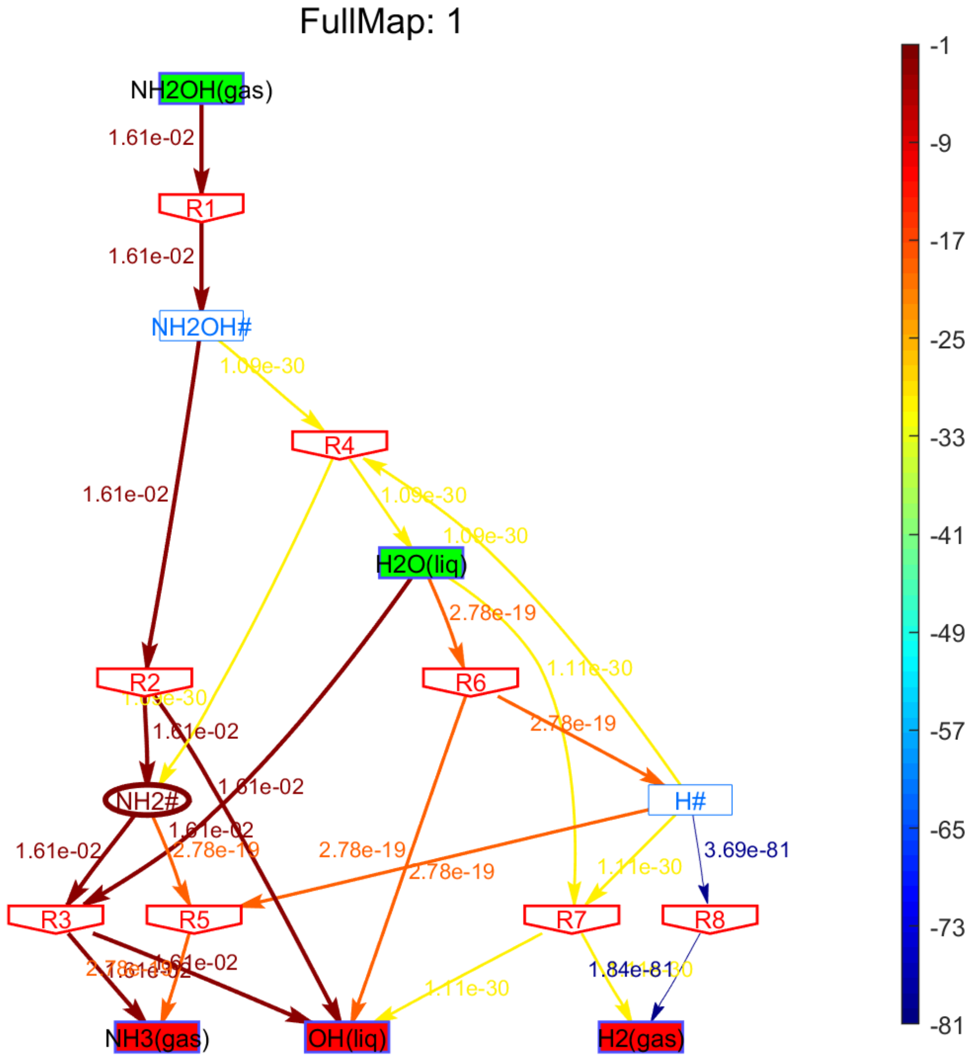

Figure S44. The full flow chart of NH_2OH reduction at NH_2 adsorption of -0.25 eV and H adsorption energy of 0.75 eV.

4. Supporting References

[1] a)G. Kresse, J. Furthmuller, *Phys. Rev. B: Condens. Matter* **1996**, 54, 11169; b)G. Kresse, J. Hafner, *Phys. Rev. B: Condens. Matter* **1993**, 47, 558.
[2] J. P. Perdew, K. Burke, M. Ernzerhof, *Physical Review Letters* **1996**, 77, 3865.
[3] S. Grimme, J. Antony, S. Ehrlich, H. Krieg, *Journal of Chemical Physics* **2010**, 132, 154104.
[4] H. J. Monkhorst, J. D. Pack, *Phys. Rev. B: Condens. Matter* **1976**, 13, 5188.
[5] a)A. Alavi, P. Hu, T. Deutsch, P. L. Silvestrelli, J. Hutter, *Phys. Rev. Lett.* **1998**, 80, 3650; b)Z. P. Liu, P. Hu, *J. Am. Chem. Soc.* **2003**, 125, 1958; c)A. Michaelides, Z. P. Liu, C. J. Zhang, A. Alavi, D. A. King, P. Hu, *J. Am. Chem. Soc.* **2003**, 125, 3704.
[6] a)K. Mathew, R. Sundararaman, K. Letchworth-Weaver, T. A. Arias, R. G. Hennig, *J. Chem. Phys.* **2014**, 140, 084106; b)K. Mathew, V. S. C. Kolluru, S. Mula, S. N. Steinmann, R. G. Hennig, *J. Chem. Phys.* **2019**, 151, 234101.
[7] H. Ogasawara, B. Brena, D. Nordlund, M. Nyberg, A. Pelmenschikov, L. G. Pettersson, A. Nilsson, *Phys. Rev. Lett.* **2002**, 89, 276102.
[8] S. Goedecker, *J. Chem. Phys.* **2004**, 120, 9911.
[9] J. Rossmeisl, E. Skúlason, M. E. Björketun, V. Tripkovic, J. K. Nørskov, *Chem. Phys. Lett.* **2008**, 466, 68.
[10] R. Tran, Z. Xu, B. Radhakrishnan, D. Winston, W. Sun, K. A. Persson, S. P. Ong, *Sci. Data* **2016**, 3, 160080.
[11] R. Tran, J. Lan, M. Shuaibi, B. M. Wood, S. Goyal, A. Das, J. Heras-Domingo, A. Kolluru, A. Rizvi, N. Shoghi, A. Sriram, F. Therrien, J. Abed, O. Voznyy, E. H. Sargent, Z. Ulissi, C. L. Zitnick, *ACS Catal.* **2023**, 13, 3066.
[12] Y.-L. Liao, B. Wood, A. Das, T. Smidt, *arXiv preprint arXiv:2306.12059* **2023**.
[13] J. K. Norskov, J. Rossmeisl, A. Logadottir, L. Lindqvist, J. R. Kitchin, T. Bligaard, H. Jonsson, *J. Phys. Chem. B* **2004**, 108, 17886.
[14] S. Ringe, C. G. Morales-Guio, L. D. Chen, M. Fields, T. F. Jaramillo, C. Hahn, K. Chan, *Nat. Commun.* **2020**, 11, 33.
[15] W. L. Holstein, M. Boudart, *J. Phys. Chem. B* **1997**, 101, 9991.
[16] C. T. Campbell, *ACS Catal.* **2017**, 7, 2770.
[17] a)L. Guo, X. Wang, L. Lu, H. Cao, Y. Dai, K. Tang, N. Zhao, F. Qi, X. Ouyang, *Appl. Surf. Sci.* **2025**, 684; b)H. Cao, H. Li, F. Liu, W. Luo, F. Qi, N. Zhao, X. Ouyang, B. Liao, *Ceram. Int.* **2022**, 48, 5476.
